# Vismodegib Exerts a Pleotropic Effect on Topotecan-Resistant and Sensitive Ovarian Cancer Cell Lines in 2D and 3D Cell Culture Models

**DOI:** 10.3390/molecules31132331

**Published:** 2026-07-02

**Authors:** Piotr Stasiak, Justyna Sopel, Artur Płóciennik, Julia Maria Lipowicz, Agnieszka Anna Rawłuszko-Wieczorek, Karolina Sterzyńska, Monika Świerczewska, Jan Korbecki, Radosław Januchowski

**Affiliations:** 1 Institute of Biological Sciences, University of Zielona Gora, 65-417 Zielona Gora, Poland; 2 The Doctoral School of Exact and Technical Sciences, University of Zielona Gora, 65-417 Zielona Gora, Poland; 3 Institute of Health Sciences, Collegium Medicum, University of Zielona Gora, 65-417 Zielona Gora, Poland; 4 Department of Histology and Embryology, Doctoral School, Poznan University of Medical Sciences, 61-701 Poznan, Poland; 5 Department of Histology and Embryology, Poznan University of Medical Sciences, 61-701 Poznan, Poland

**Keywords:** ovarian cancer, multidrug resistance, Vismodegib, breast cancer resistance protein (BCRP), 3D cell culture model

## Abstract

Ovarian cancer remains a leading cause of gynecological cancer-related mortality. Although the standard therapy is usually effective at first, the development of multidrug resistance (MDR) significantly limits the efficacy of the treatment. Topotecan (TOP) is a drug commonly used in second-line therapy. However, the cancer cells eventually become immune to its cytotoxic effect, as they start to produce ATP-binding cassette (ABC) transporters, proteins facilitating drug removal. Breast cancer resistance protein (BCRP) is one such protein, and TOP is its known substrate. Vismodegib, a Sonic Hedgehog pathway (Shh) inhibitor, has demonstrated potential anticancer activity in various malignancies. This study aims to evaluate the effects of Vismodegib treatment on both topotecan-sensitive and -resistant ovarian cancer cell lines, cultured in two-dimensional (2D) and three-dimensional (3D) conditions, as well as the potential of Vismodegib treatment to reverse the acquired drug resistance. Our results suggest that Vismodegib re-sensitizes TOP-resistant cells in both 2D and 3D models. However, further research is needed to evaluate its usefulness in ovarian cancer treatment.

## 1. Introduction

Ovarian cancer is one of the most challenging diseases in gynecologic oncology and ranks eighth in terms of mortality among women’s cancers. The majority of patients with ovarian cancer are diagnosed in advanced disease stages, mainly in FIGO III or IV [1]. The standard treatment in ovarian cancer is surgical resection following platinum-based chemotherapy. The first-line chemotherapy administered after surgery is composed of platinum- and taxane-based drugs [2]. Patients who are primarily resistant to this therapy constitute only 5%, but unfortunately, drug resistance develops during treatment in the majority of patients [2,3]. Second-line chemotherapy for patients who are primarily sensitive to platinum includes platinum-based drugs and other agents [4]. In patients with a poor response to platinum, second-line chemotherapy typically consists of drugs such as gemcitabine, topotecan (TOP), and doxorubicin (DOX) [5,6]. However, response to second-line chemotherapy is typically low (15–35%).

Two factors are primarily responsible for low response to chemotherapy. The first is the late diagnosis of the cancer and its advanced stage. The second is primary drug resistance or drug resistance developed during treatment. In most cases, the drug resistance has a characteristic of multidrug resistance (MDR), where cancer cells are cross-resistant to different cytotoxic drugs. The main proteins responsible for MDR are drug transporters from the ABC family. They utilize energy from ATP hydrolysis and actively efflux drugs from cells [7]. Among them, the pivotal roles in cancer drug resistance are played by glycoprotein P (P-gp) and breast cancer resistance protein (BCRP) [7]. Their expression is associated with reduced progression-free survival in patients with ovarian cancer [8].

The BCRP protein is encoded by the *ABCG2* gene. It comprises 16 exons and is located on chromosome 4q22 [9]. It is a half transporter with a molecular mass of 60 kDa, with mass increasing to about 70 kDa after N-glycosylation at position Asn59 [10]. Transporter activity is achieved after its homodimerization [9,10]. Among about 200 BCRP substrates, we can distinguish different cytotoxic drugs such as Methotrexate (MTX), mitoxantrone (MIT), and TOP used in second-line chemotherapy of ovarian cancer [9,11]. Overexpression of BCRP is observed in many drug-resistant cancers, such as liver [12], lung [12], breast [13], and ovarian cancer [14]. In drug-resistant cell lines, overexpression of BCRP is induced mainly after MIT or TOP treatment [11], and it was observed by our team in all TOP-resistant ovarian cancer cell lines [15].

Among other factors that play a role in the resistance of tumors to chemotherapy is the architecture of tumor tissue. High cell density and the expression of extracellular matrix (ECM) proteins by tumor cells and tumor-associated fibroblasts (CAFs) effectively limit drug diffusion [16,17]. In particular, large molecules, such as Metotrexate (MTX) or paclitaxel (PAC), have limited ability to diffuse through dense cellular structures [18,19]. Additionally, ECM components can activate intracellular signaling pathways by binding to cell-surface receptors, thereby causing cell adhesion-mediated drug resistance (CAM-DR) [20].

According to the cancer stem cell (CSC) model of cancer progression, CSCs play the most important role in the development of drug resistance [21]. CSCs are a small population of cells present in tumors that are primarily resistant to radiotherapy and chemotherapy [22]. Resistance to chemotherapeutic drugs is in part due to their high expression levels of drug transporters such as P-gp and/or BCRP, and they also possess other drug resistance mechanisms [23]. Thus, CSCs survive chemotherapy and then differentiate into CSCs and more differentiated cancer cells that are still resistant to chemotherapeutic drugs [24]. Additionally, CSCs contribute to tumor recurrence, metastasis, multidrug resistance, and radioresistance through their ability to remain in the G0 phase and differentiate into multiple cell subtypes [25].

The biology of CSCs is regulated by specific signaling pathways designated as CSC signaling pathways, among which Notch, Wnt/β-catenin, and Sonic Hedgehog (SHH) play a central role.

The SHH signaling pathway is crucial in the proliferation and differentiation of adult stem cells and also influences embryogenesis, cellular development, epithelial–mesenchymal transition (EMT), and cancer stem cell (CSC) maintenance by regulating cellular metabolism, self-renewal, migration, and invasion [26,27].

Canonical activation of the pathway occurs through the binding of the SHH glycoprotein ligand to the 12-transmembrane protein Patched (PTCH1), resulting in its inactivation. This, in turn, results in derepression of the 7-transmembrane protein SMO and its translocation from the endosomal membrane to the plasma membrane [27,28]. At this point, complete SMO activation occurs. Active SMO inhibits PKA activity, preventing phosphorylation of the Sufu/GSK3β/Gli complex, which results in its dissociation and releases the GLI family transcription factors (GLI1, GLI2, GLI3). These factors can be transported to the cell nucleus and regulate gene transcription [27,28,29].

Among genes regulated by SHH pathway, we can distinguish SHH pathway genes that are regulated via a positive feedback loop (e.g., *GLI1*, *PTCH1*), genes encoding ABC transporters (*MDR1* (*ABCB1*), *BCRP* (*ABCG2*)) [30], genes regulating cell cycle (*CCND1/2*, *CCNE*, *Cdk6*, *N-Myc*) [31] and genes that regulate the EMT (*Cdh2*, *VIM*, *FN1*, *ACTA2*; *CDH1*) [32].

Abnormalities in SHH signaling are an important factor in the development of cancer [28,33], influencing processes such as tumorigenesis, progression, metastasis, and drug resistance [34]. The SHH signaling abnormalities have been described in prostate, lung, pancreatic, breast, colon, hepatocellular, bladder, and ovarian cancers [34].

As an important factor in cancer development and progression, the SHH pathway seems to be very attractive in anticancer therapy. One of the drugs targeting the SHH pathway is Vismodegib (VIS). VIS is a selective, second-generation inhibitor of the SHH pathway developed by Roche/Genentech/Curis [35,36]. It is also known as GDC-0449, HhAntag691, or 2-chloro-N-[4-chloro-3-pyridin-2-yl-phenyl]-4-methanesulfonyl benzamide. The molecular weight of the compound is 421.30 g/mol [36]. VIS has been developed as a cyclopamine derivative, with greater potency and more favorable pharmacological properties. The advantage of VIS is its ability to cross the blood–brain barrier after oral administration [37]. At the molecular level, it is a selective antagonist of the SMO protein. Binding the inhibitor to SMO inhibits further activation of the SHH pathway and, consequently, downstream target genes [38].

Vismodegib was approved by the FDA as an inhibitor of the SHH pathway in 2012 and by the European Commission in 2013 for the treatment of basal cell carcinoma (BCC) [39]. VIS is used to treat advanced or surgically unresectable BCC, as well as to treat metastatic BCC [40]. Moreover, clinical trials are underway using VIS with gemcitabine hydrochloride, nab-paclitaxel, or other chemotherapeutics in the treatment of BCC, glioblastoma, and pancreatic adenocarcinoma [41,42].

In addition to studies on the effectiveness of VIS on primary BCC tumors and their metastases, studies on other cancers are also ongoing. Yeh et al. showed that in non-small cell lung cancer (NSCLC), the combination of PAC with VIS increased its cytotoxicity [43]. Additionally, co-treatment of medulloblastoma (MB) with VIS and an EZH2 inhibitor produced greater inhibition of MB growth than monotherapy with either drug [44]. The use of PR1-724 and VIS in the treatment of head and neck squamous cell carcinoma (HNSCC) cells resulted in cell arrest in the G1/G0 phase, reduced cell migration and decreased the transcript levels of CSC markers [45]. Treatment of human prostate cancer with VIS increased cellular apoptosis and tumor volume in a mouse xenograft model [46]. VIS also inhibited EMT transition in castration-resistant prostate cancer (CRPC) [46], inhibited proliferation, invasion, and mammosphere formation in breast cancer cells, and induced cell apoptosis [47]. Blocking the SHH pathway by cyclopamine in adenocarcinoma and prostate cancer cells caused a decrease in Gli-1 expression, which in turn led to a decrease in MDR1 and BCRP expression and decreased resistance to docetaxel, MTX, and etoposide [30].

During research on VIS, it has been observed that the second mechanism of VIS activity is related to its influence on drug transport. VIS prevents the action of ATP-binding cassette (ABC) transporters, making it an inhibitor of the ABC transporters ABCG2/BCRP and ABCB1/P-gp, as well as a mild inhibitor of ABCC1/MRP1, all of which are responsible for MDR [48]. In cells overexpressing BCRP, the addition of VIS resulted in increased retention of the fluorescent substrate BODIPY-prazosin. VIS also increased the retention of Calcein-AM in cells overexpressing P-gp or MRP1. The study also showed that the addition of VIS during colchicine treatment of Madin–Darby canine kidney II (MDCKII) cells overexpressing P-gp and MRP1 sensitized the cells to colchicine. Co-treatment with VIS and either MTX, TOP, or SB-38 also sensitized human non-small-lung carcinoma cells to the treatment [48].

Two-dimensional (2D) cell cultures are a standard model used to study MDR mechanisms. However, they do not reflect the conditions prevailing within a cancer tumor, particularly the cellular structure and expression of extracellular matrix (ECM) molecules, which influence limited drug diffusion [19]. In contrast, the 3D spheroids model reflects the mechanisms of tissue resistance, which is related to the histological structure of the tumor (high cell density) or the expression of ECM molecules [49]. Our previous experiments have shown that cells cultured as spheroids have significantly greater resistance to chemotherapeutics than those cultured as monolayers [19,50].

To investigate the effect of VIS on drug resistance in ovarian cancer, we used the A2780 drug-sensitive ovarian cancer cell line, and cell lines resistant to TOP (A2780TR1 and A2780TR2), a drug used in the second line of ovarian cancer therapy, which are characterized by high levels of BCRP expression [51]. Both a 2D model and a 3D spheroid model formed under non-adhesive conditions were used in the study to better reflect the tumor environment. A deep analysis of the VIS effect on drug resistance in 2D and 3D models of drug resistance is a subject of study presented in this manuscript.

## 2. Results

### 2.1. Characterization of Drug-Sensitive and Resistant Cell Lines

Before starting experiments with VIS, we characterized the drug-sensitive and resistant cell lines to determine the levels of SHH pathway genes, as well as other genes associated with drug resistance and the expression of related proteins.

#### 2.1.1. Expression Analysis of BCRP in Ovarian Cancer Cells

**Figure 1:**
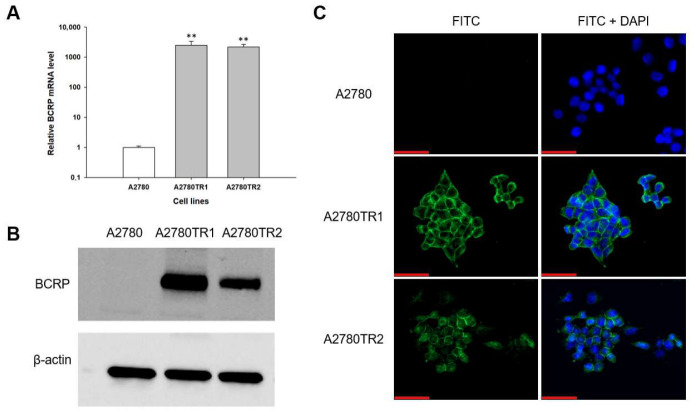
(**A**) Expression analysis of BCRP transcript (Q-PCR) in the A2780 and TOP-resistant cell sublines. The figure presents the relative gene expression in the resistant cell lines (gray bars) with respect to that in the sensitive cell line (white bars), which has been assigned a value of 1. The values were considered significant at ** *p* < 0.01. (**B**) BCRP protein expression analysis in the A2780 and TOP-resistant cell lines. The cellular proteins were separated using a 4–20% mini-PROTEAN TGX gel, using SDS-PAGE electrophoresis, and transferred to a nitrocellulose membrane, which was then immunoblotted with either primary Ab or HRP-conjugated secondary Ab. A primary anti-β-actin Ab was used as a loading control for the cell lysates. (**C**) BCRP immunofluorescence in the A2780 and TOP-resistant cell sublines. BCRP was detected using the anti-BCRP antibody and Alexa Fluor^®^488-conjugated secondary antibody (green). To visualize the cell nuclei, the cells were mounted with a DAPI-containing mounting medium (blue). Objective 40×. Scale bar = 50 μm.

The expression of the BCRP protein, which is the most important ABC transporter responsible for TOP resistance, was checked in the studied cell lines. The qPCR results show that TOP-resistant cell lines have about 5000-fold higher levels of BCRP gene expression compared to the chemotherapy-sensitive cell line (Figure 1A), *p* < 0.01. Analysis of BCRP protein levels, by Western blot, did not detect BCRP protein in the A2780 cell line, while the protein was present in the A2780TR1 and A2780TR2 cell lines (Figure 1B). Moreover, immunofluorescence staining showed that the BCRP protein is present only in TOP-resistant cell lines and localized mainly in the cell membrane (Figure 1C).

#### 2.1.2. Expression Analysis of ALDH1A1 in Ovarian Cancer Cells

**Figure 2:**
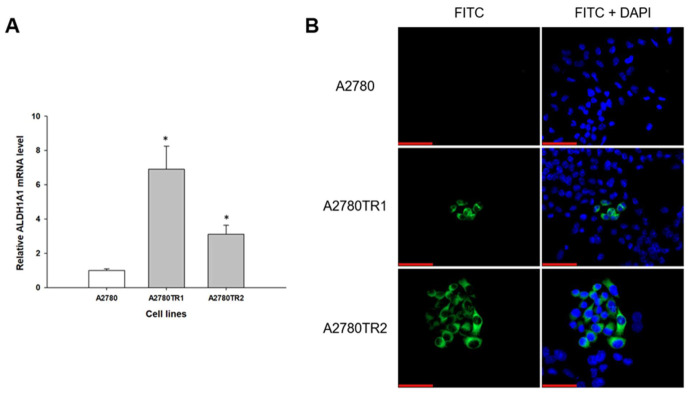
(**A**) Expression analysis of ALDH1A1 transcript (Q-PCR) in the A2780 and TOP-resistant cell sublines. The figure presents the relative gene expression in the resistant cell lines (gray bars), with respect to that in the sensitive cell line (white bars), which has been assigned a value of 1. The values were considered significant at * *p* < 0.05. (**B**) ALDH1A1 immunofluorescence in the A2780 and TOP-resistant cell sublines. ALDH1A1 was detected using the anti-ALDH1A1 antibody and Alexa Fluor^®^488-conjugated secondary antibody (green). To visualize the cell nuclei, the cells were mounted with a DAPI-containing mounting medium (blue). Objective 40×. Scale bar = 50 μm.

Next, the expression ALDH1A1, a marker of CSCs, was investigated. We observed 7-fold higher ALDH1A1 transcript expression in the A2780TR1 cell line and 3-fold higher expression in the A2780TR2 cell line compared with the TOP-sensitive cell line (Figure 2A), *p* < 0.05. Immunofluorescence staining showed that A2780 cells do not express the ALDH1A1 protein. However, in TOP-resistant cell lines, we observed cell populations with and without ALDH1A1 expression (Figure 2B).

#### 2.1.3. Expression Analysis of PTPRK in Ovarian Cancer Cells

**Figure 3:**
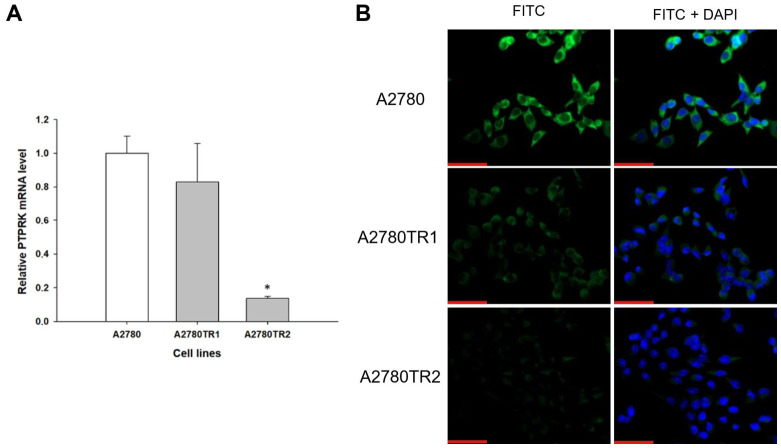
(**A**) Expression analysis of PTPRK transcript (Q-PCR) in the A2780 and TOP-resistant cell sublines. The figure presents the relative gene expression in the resistant cell lines (gray bars) with respect to that in the sensitive cell line (white bars), which has been assigned a value of 1. The values were considered significant at * *p* < 0.05. (**B**) PTPRK immunofluorescence in the A2780 and TOP-resistant cell sublines. PTPRK was detected using the anti-PTPRK antibody and Alexa Fluor^®^488-conjugated secondary antibody (green). To visualize the cell nuclei, the cells were mounted with a DAPI-containing mounting medium (blue). Objective 40×. Scale bar = 50 μm.

The expression of PTPRK (Protein Tyrosine Phosphatase Receptor Type K) was investigated in the tested cell lines (Figure 3). Analysis of the PTPRK gene expression in the studied cell lines showed that A2780TR1 cells have gene expression profiles similar to those of the TOP-sensitive cell line. However, the A2780TR2 cell line has significantly lower PTPRK gene expression than the other lines (Figure 3A), *p* < 0.05. Immunofluorescence analysis showed decreased PTPRK fluorescence in both TOP-resistant cell lines compared with the A2780 cell line (Figure 3B).

#### 2.1.4. Expression Analysis of pTYR in Ovarian Cancer Cells

**Figure 4:**
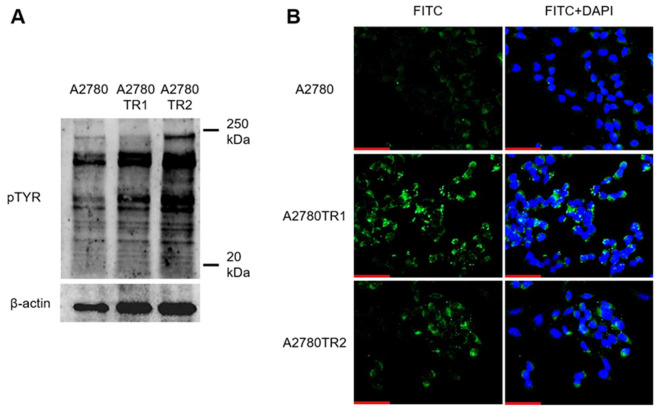
(**A**) Total pTYR protein expression analysis in the A2780 and TOP-resistant cell lines. The cellular proteins were separated using a 4–20% mini-PROTEAN TGX gel, using SDS-PAGE electrophoresis, and transferred to a PVDF membrane, which was then immunoblotted with either primary Ab or HRP-conjugated secondary Ab. A primary anti-β-actin Ab was used as a loading control. (**B**) Immunofluorescence visualization of pTYR expression in the A2780 and TOP-resistant cell lines. pTYR was detected using the anti-pTYR antibody and MFP488-conjugated secondary antibody (green). Cell nuclei were stained with DAPI (blue). Objective 40×. Scale bar = 50 μm.

Analysis of pTYR protein expression in the tested cell lines showed that TOP-resistant cell lines had higher total tyrosine phosphorylation levels than the A2780 cell line. A difference in pTYR expression is also observed between the TOP-resistant cell lines. Specifically, the A2780TR2 cell line has increased expression of the pTYR protein compared to A2780TR1 (Figure 4A). In immunofluorescence analysis, we also observed increased fluorescence in both TOP-resistant cell lines (Figure 4B).

#### 2.1.5. Analysis of the Expression of Selected Genes Involved in the SHH Signaling Pathway

**Figure 5:**
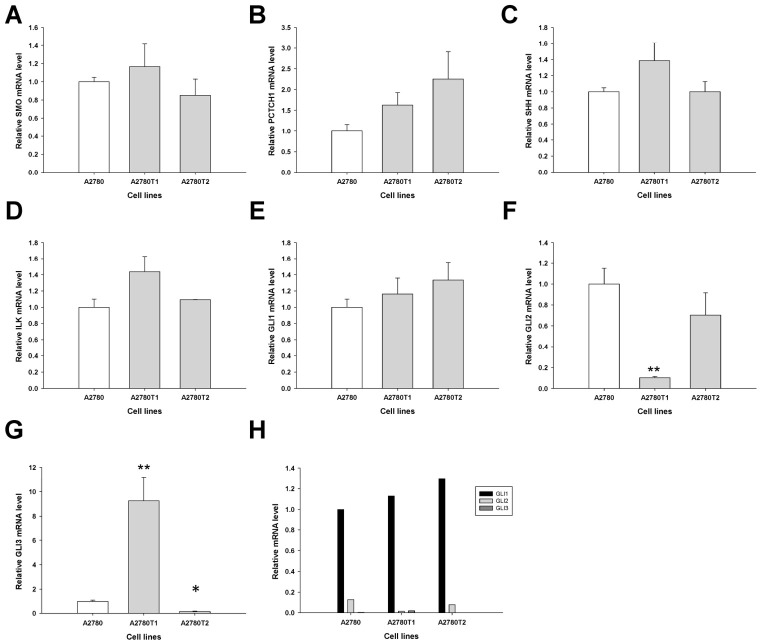
Analysis of gene expression in the sensitive line—A2780—and TOP-resistant lines—A2780TR1 and A2780TR2. The SMO (**A**), PCTCH1 (**B**), SHH (**C**), ILK (**D**), GLI1 (**E**), GLI2 (**F**), and GLI3 (**G**) genes were analyzed by qPCR. (**H**)—relative expression analysis of different GLI isoforms in drug-sensitive A2780 and TOP-resistant A2780TR1 and A2780TR2 cell lines in relation to the expression level of the GLI1 isoform set as 1 in line A2780. The figure presents the relative gene expression in the resistant cell lines (gray bars) with respect to that in the sensitive cell line (white bars), which has been assigned a value of 1. The values were considered significant at * *p* < 0.05, ** *p* < 0.01.

Analysis of the expression of selected genes participating in the SHH pathway was performed in the tested cell lines. The expression of SMO, PCTCH1, SHH, ILK, and GLI1 genes does not differ significantly between the sensitive cell line and the TOP-resistant cell lines (Figure 5A–E). However, the expression of the GLI2 gene was about 10-fold lower in the A2780TR1 cell line than in the TOP-sensitive cell line (Figure 5F), *p* < 0.01. In contrast, expression of the GLI3 gene was about 10-fold higher for the A2780TR1 cell line in comparison to the A2780 cell line, *p* < 0.01, while for the A2780TR2 cell line, its expression decreased approximately two-fold compared to the A2780 cell line (Figure 5G), *p* < 0.05.

#### 2.1.6. The Response of Drug-Sensitive and Resistant Cell Lines to VIS Treatment in the 2D Model

**Figure 6:**
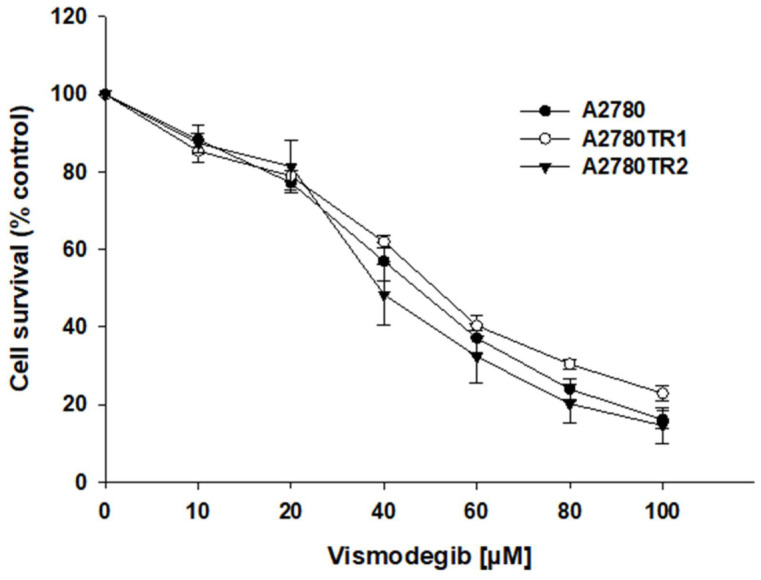
VIS presents concentration-dependent cytotoxicity. Cell lines were cultured in 96-well plates and treated with selected concentrations of VIS for 72 h. Next, the MTT cell survival assay was performed to determine cell viability. The cell viability assay was expressed as a percentage of the untreated control (mean ± SEM).

The effect of SHH pathway inhibitor (SMO protein inhibitor), Vismodegib, on the viability of ovarian cancer cells was tested. We observed very similar, concentration-dependent responses to VIS treatment in drug-sensitive and both drug-resistant cell lines (Figure 6). Based on the response curve, the IC_25_ and IC_50_ values of VIS were determined for the tested cell lines (Table 1). The IC_25_ and IC_50_ values for the TOP-sensitive cell line were 24.2 µM and 46.1 µM, respectively. The TOP-resistant cell lines showed quite similar IC_25_ and IC_50_ values to the sensitive cell line. Specifically, the IC_25_ for the A2780TR1 cell line was 24.7 µM, and the IC_50_ was 51.5 µM. For the A2780TR2 cell line, the IC_25_ was 23.7 µM, and the IC_50_ was 42.5 µM.

### 2.2. The Effect of VIS on the Drug Resistance Mechanism

Next, we wanted to establish the molecular mechanism of VIS. Cell lines were cultured for 72 h in the absence or presence of VIS at concentrations of 10 µM, 25 µM, and 50 µM, corresponding to the IC_10_, IC_25_, and IC_50_ values of VIS.

#### 2.2.1. Apoptosis and Necrosis

**Figure 7:**
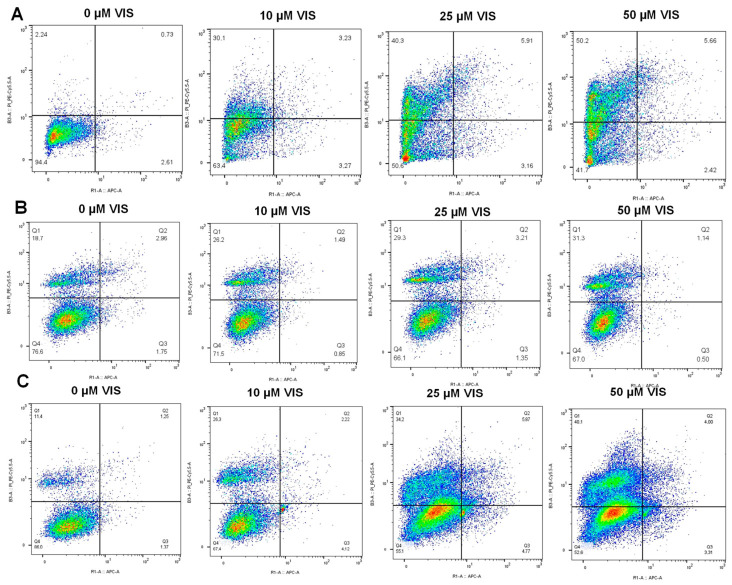
Analysis of cell apoptosis/necrosis after 72 h of VIS treatment. The apoptosis test was performed by analyzing the number of cells stained with Annexin-V and PI. Results showed the percentage of apoptotic, live, and necrotic cells (**A**) A2780, (**B**) A2780T1, (**C**) A2780T2.

We investigated the effect of VIS on apoptosis and necrosis after 72 h of treatment in the A2780, A2780TR1, and A2780TR2 cell lines. In all cell lines, we observed a concentration-dependent increase in the percentage of necrotic cells. However, the number of early apoptotic and late apoptotic cells did not change significantly after VIS treatment (Figure 7A–C).

#### 2.2.2. Effect of VIS Treatment on pTYR Level

**Figure 8:**
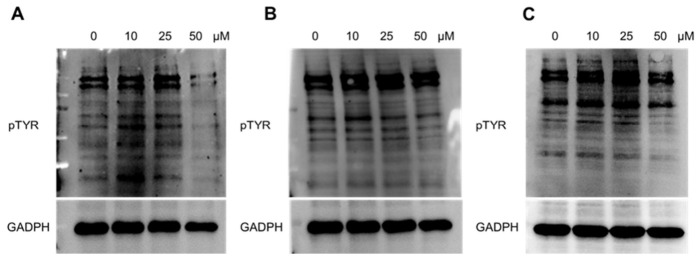
Effect of VIS on the expression of p-TYR in cell lines A2780 (**A**), A2780TR1 (**B**), and A2780TR2 (**C**). Cell lines were cultured in the absence or presence of VIS at concentrations of 10, 25, and 50 µM for 72 h in 2D in a monolayer. The cellular proteins were separated using 7% nitrocelluose and transferred to a nitrocellulose membrane, which was then immunoblotted with either primary anti-pTyr Ab or HRP-conjugated secondary Ab. A primary anti-GADPH Ab was used as a loading control.

In all cell line lysates, we observed similar levels of pTYR in cells treated with VIS at 10 µM and 25 µM, as well as in the control sample. However, treatment with VIS at 50 µM noticeably decreased total pTYR levels in the A2780 and A2780TR2 cell lines, with a minimal effect in the A2780TR1 cell line (Figure 8).

#### 2.2.3. The Effect of Co-Treatment with VIS and TOP on Cell Viability

**Figure 9:**
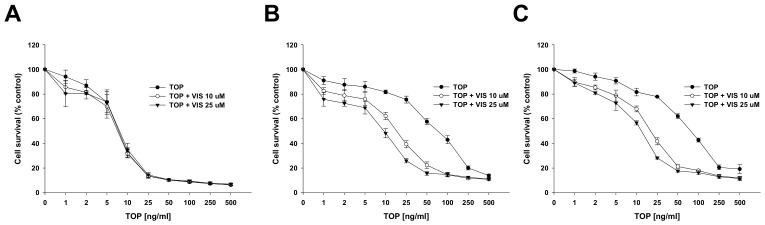
VIS sensitizes drug-resistant cell lines to TOP in vitro. Cell lines sensitive (**A**) and resistant to TOP—A2780TR1 (**B**) and A2780TR2 (**C**)—were seeded into 96-well plates. The cells were treated with increasing concentrations of TOP in the absence or presence of VIS at 10 µM and 25 µM. After 72 h-long incubation, cell viability was determined using the MTT assay.

Next, we were interested in whether VIS would change sensitivity to TOP treatment in the investigated cell lines. All cell lines were treated with increasing concentrations of TOP, in the absence or presence of VIS at 10 µM or 25 µM, corresponding to IC_10_ and IC_25_ values. In the A2780 cell line, we did not observe any effect of VIS on TOP resistance (Figure 9A) (Table 2). The IC_50_ for TOP alone was 7.68 ng/mL; in the presence of 10 µM VIS, it was 7.23 ng/mL, and in the presence of 25 µM VIS, it was 7.94 ng/mL.

However, treatment of TOP-resistant cells with VIS resulted in a decrease in their sensitivity to TOP, and the effect was VIS concentration-dependent (Figure 9B,C). For the A2780TR1 cell line treated with TOP, the IC_50_ value decreased by 4.20-fold in the presence of 10 µM VIS (IC_50_ = 75.5 ng/mL vs. IC_50_ = 17.9 ng/mL, *p* < 0.01) and 7.40-fold in the presence of 25 µM VIS (IC_50_ = 75.5 ng/mL vs. IC_50_ = 10.2 ng/mL, *p* < 0.01). For the A2780TR2 cell line treated with TOP, the IC_50_ value decreased by 3.98-fold in the presence of 10 µM VIS (IC_50_ = 81.1 ng/mL vs. IC_50_ = 20.4 ng/mL, *p* < 0.001) and 6.03-fold in the presence of 25 µM VIS (IC_50_ = 81.1 ng/mL vs. IC_50_ = 13.4 ng/mL, *p* < 0.001).

#### 2.2.4. Effect of Vismodegib on BCRP Expression

**Figure 10:**
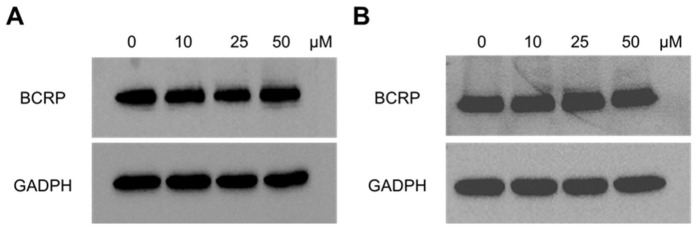
Effect of VIS on the expression of BCRP protein in cell lines A2780TR1 (**A**) and A2780TR2 (**B**). Cell lines were grown in the absence or presence of VIS at concentrations of 10 µM, 25 µM, and 50 µM for 72 h. The cellular proteins were separated using a 4–20% mini-PROTEAN TGX gel, using SDS-PAGE electrophoresis, and transferred to a nitrocellulose membrane, which was then immunoblotted with either primary anti-BCRP Ab or HRP-conjugated secondary Ab. A primary anti-GADPH Ab was used as a loading control.

As the BCRP protein is the primary protein responsible for TOP resistance, we focused on the effect of VIS on its expression. Notably, 72 h treatment with VIS at concentrations of 10, 25, and 50 µM did not change BCRP protein levels in any of the resistant cell lines (Figure 10A,B).

#### 2.2.5. The Effect of 72 h Treatment with VIS on BCRP Protein Activity

As we did not observe the effect of VIS on BCRP protein levels, we were interested in whether long-term VIS treatment could change BCRP protein activity. We investigated this possibility using a fluorescent microscope and flow cytometry techniques, and H33342 and Mitoxantrone (MIT) as BCRP substrates. In all these experiments, the cells were treated with VIS for 72 h, although VIS was washed from the cell culture medium before microscope or flow cytometry experiments.

**Figure 11:**
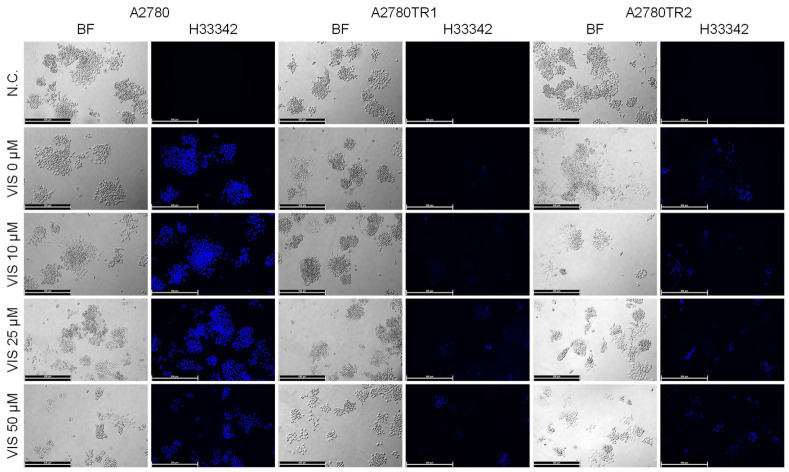
Fluorescent microscopy analysis of H33342 accumulation (blue) in drug-sensitive A2780 cell line and TOP-resistant cell lines A2780TR1 and A2780TR2. Cell lines were treated with VIS at concentrations of 10, 25, and 50 µM for 72 h. Next, the VIS was washed out of the cell culture medium, and fluorescence imaging with bright-field (BF) visualization was performed. Scale bar = 500 µm.

**Figure 12:**
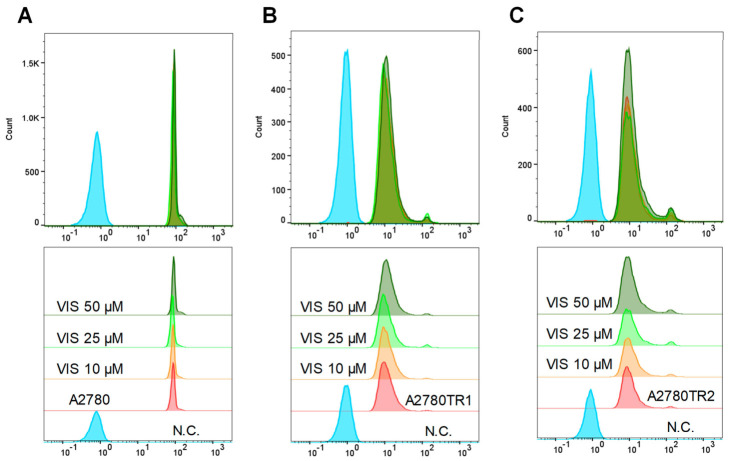
Flow cytometry analysis of intracellular accumulation of H33342 in a drug-sensitive A2780 cell line (**A**) and TOP-resistant cell lines A2780TR1 (**B**) and A2780TR2 (**C**). Cell lines were treated with VIS at concentrations of 10, 25, and 50 µM for 72 h. Next, the VIS was washed out of the cell culture medium, and flow cytometry analysis was performed. The diagrams of fluorescence intensity show the fluorescence intensity in the untreated cell line (red) and cell lines treated with VIS at concentrations of 10 µM (orange), 25 µM (green), and 50 µM (dark green). N.C.—negative control without MIT (blue).

Fluorescent microscope (Figure 11) and flow cytometry (Figure 12) analysis of H33342 accumulation showed no significant effect of VIS treatment on H33342 accumulation in all cell lines tested. The sensitive cell line shows strong accumulation of H33342 regardless of the VIS concentration used. In contrast, the A2780TR1 and A2780TR2 cell lines exhibit only low levels of accumulation, which are also independent of the inhibitor concentration.

**Figure 13:**
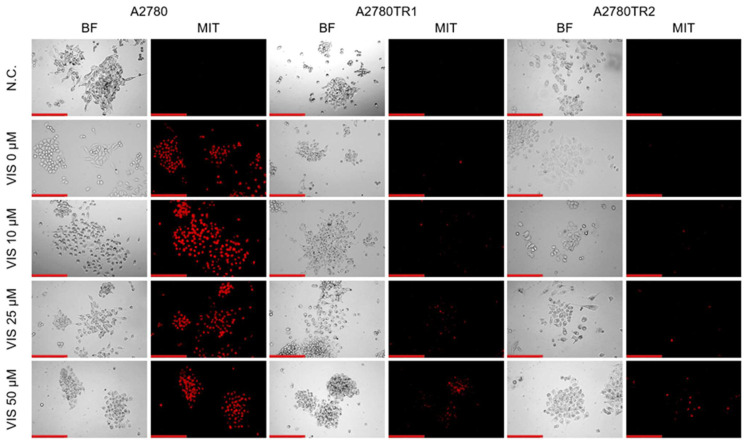
Fluorescent microscopy analysis of MIT accumulation (red) in drug-sensitive A2780 cell line and TOP-resistant cell lines A2780TR1 and A2780TR2. Cell lines were treated with VIS at concentrations of 10, 25, and 50 µM for 72 h. Next, the VIS was washed from the cell culture medium, and fluorescence analysis and bright field visualization (BF) were performed. Scale bar = 200 µm.

**Figure 14:**
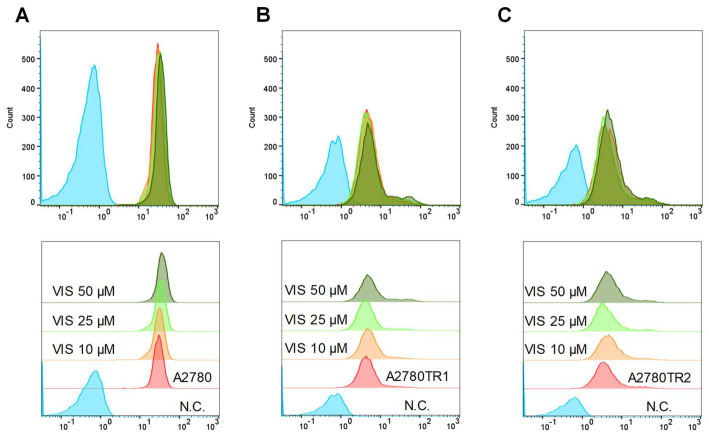
Flow cytometry analysis of intracellular accumulation of MIT in a drug-sensitive A2780 cell line (**A**) and TOP-resistant cell lines A2780TR1 (**B**) and A2780TR2 (**C**). Cell lines were treated with VIS at concentrations of 10, 25, and 50 µM for 72 h. Next, the VIS was washed from the cell culture medium, and flow cytometry analysis was performed. The diagrams of fluorescence intensity show the fluorescence intensity in the untreated cell line (red) and cell lines treated with VIS at concentrations of 10 µM (orange), 25 µM (green), and 50 µM (dark green). N.C.—negative control without MIT (blue).

Examination of MIT accumulation in cell lines also showed no significant differences after VIS treatment (Figure 13 and Figure 14). We observed a similar level of MIT accumulation in the A2780 cell line in the presence and absence of different VIS concentrations (Figure 13 and Figure 14A). In TOP-resistant cell lines, we did not observe MIT accumulation in the untreated cell population. However, treatment of cells with higher VIS concentration resulted in MIT accumulation is very small population of cells (Figure 13). However, this was not confirmed by flow cytometry analysis (Figure 14B,C).

#### 2.2.6. The Effect of VIS on the Activity of the BCRP Protein

Next, we wanted to establish whether the presence of VIS in cell culture medium would change the activity of the BCRP protein. In all these experiments, the cells were pretreated with different VIS concentrations for one hour, and next, H33342 and MIT accumulation was investigated as before, although VIS was present during the incubation with fluorescent compounds and washed out just before the imaging was carried out.

**Figure 15:**
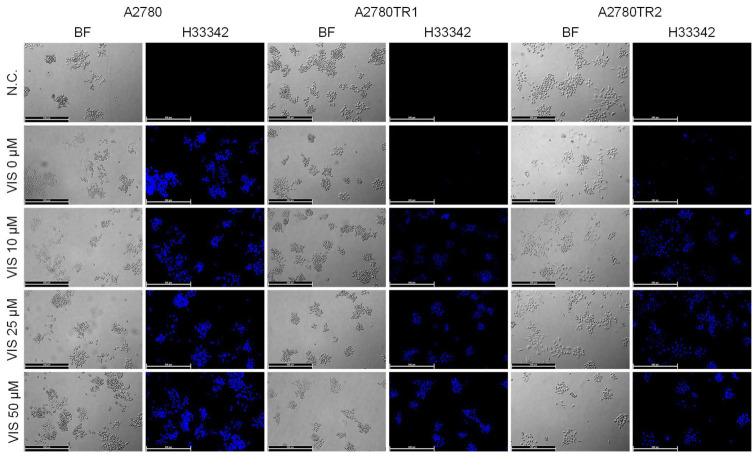
Fluorescent microscopy analysis of H33342 accumulation (blue) in drug-sensitive A2780 cell line and TOP-resistant cell lines A2780TR1 and A2780TR2 in the absence or presence of Vismodegib (VIS) at concentrations of 10, 25, and 50 µM. Cells were pretreated with VIS for one hour, and next, a fluorescence analysis and bright field visualization (BF) were performed. Scale bar = 500 µm.

**Figure 16:**
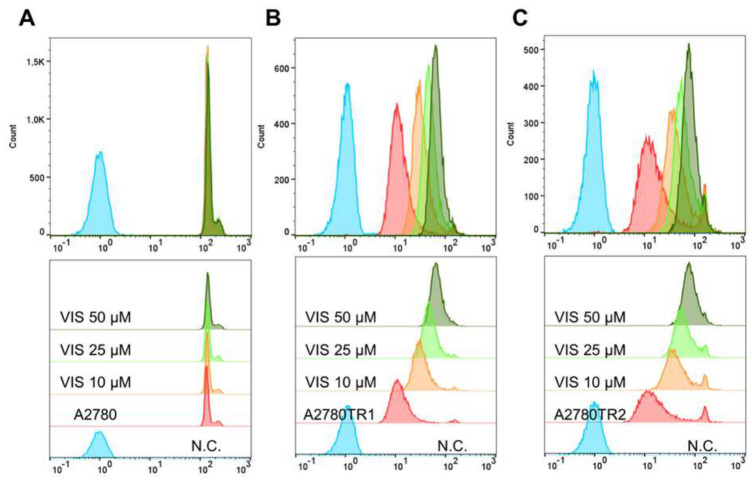
Flow cytometry analysis of intracellular accumulation of H33342 in a drug-sensitive A2780 cell line (**A**) and TOP-resistant cell lines A2780TR1 (**B**) and A2780TR2 (**C**). Cell lines were pretreated with VIS for one hour, and then, the flow cytometry analysis was performed. The diagrams of fluorescence intensity show the fluorescence intensity in the untreated cell line (red) and cell lines treated with VIS at concentrations of 10 µM (orange), 25 µM (green), and 50 µM (dark green). N.C.—negative control without H33342 (blue).

In the TOP-sensitive cells, we observed a high level of H33342 accumulation in the absence and presence of VIS (Figure 15 and Figure 16A). In contrast, a lack of H33342 accumulation was observed in both TOP-resistant cell lines without VIS (Figure 15 and Figure 16B,C). After VIS treatment, we observed accumulation of H33342 in all cells (Figure 15). Flow cytometry experiments showed that the effect of VIS on BCRP protein activity was concentration-dependent (Figure 16B,C).

**Figure 17:**
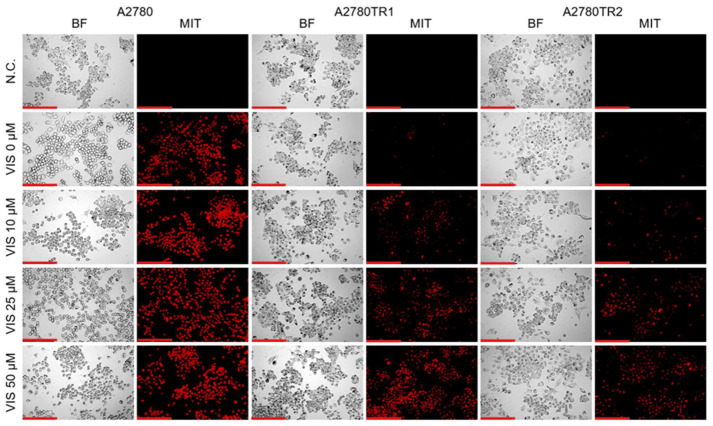
Fluorescent microscopy analysis of MIT accumulation (red) in drug-sensitive A2780 cell line and TOP-resistant cell lines A2780TR1 and A2780TR2 in the absence or presence of Vismodegib (VIS) at concentrations of 10, 25, and 50 µM. Cells were pretreated with VIS for one hour, and then, fluorescence imaging and bright field visualization (BF) were performed. Scale bar = 200 µm.

**Figure 18:**
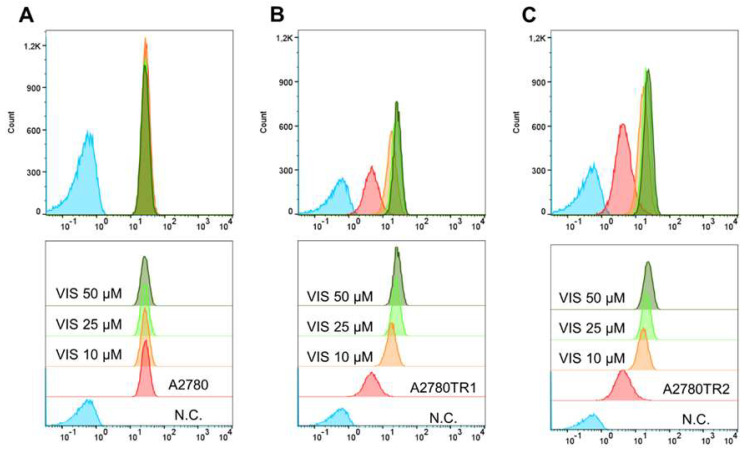
Flow cytometry analysis of intracellular accumulation of MIT in a drug-sensitive A2780 cell line (**A**) and TOP-resistant cell lines A2780TR1 (**B**) and A2780TR2 (**C**). Cell lines were pretreated with VIS for one hour, and then, the flow cytometry analysis was performed. The diagrams of fluorescence intensity show the fluorescence intensity in the untreated cell line (red) and cell lines treated with VIS at concentrations of 10 µM (orange), 25 µM (green), and 50 µM (dark green). N.C.—negative control without MIT (blue).

To confirm the effect of VIS on BCRP protein activity, analysis of MIT accumulation was also performed. In A2780 cell lines, we observed a high level of MIT accumulation regardless of the absence or presence of VIS (Figure 17 and Figure 18A). In contrast, we did not observe MIT accumulation in both resistant cell lines in the absence of VIS. Treatment with VIS resulted in concentration-dependent accumulation of MIT in TOP-resistant cell lines (Figure 17 and Figure 18B,C).

#### 2.2.7. The Effect of Co-Treatment of Ovarian Cancer Cells with VIS and TOP in the 3D Model

**Figure 19:**
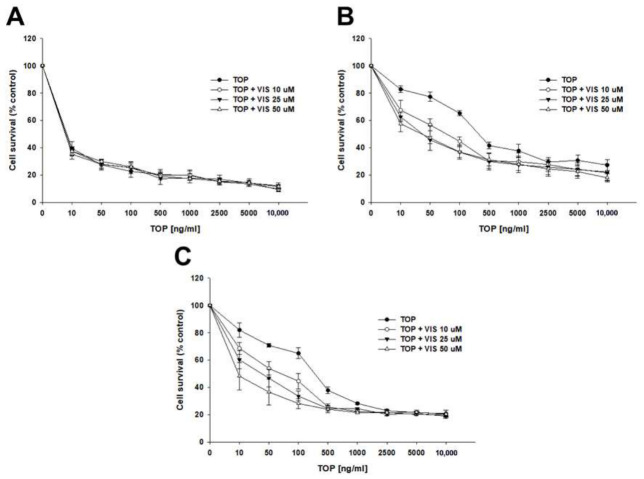
VIS sensitizes drug-resistant cell lines to chemotherapy in vitro. Cell lines: sensitive (**A**) and resistant to TOP—A2780TR1 (**B**) and A2780TR2 (**C**)—were seeded in 96-well plates. After spheroid formation, the cells were treated for 72 h with increasing concentrations of TOP in the absence or presence of VIS at concentrations of 10 µM, 25 µM, and 50 µM. After 72 h of treatment, cell viability was determined with the MTT assay.

The 2D cell culture model is an adequate tool to study drug resistance mechanisms at a single-cell level. However, it does not reflect all of the drug resistance mechanisms present in the tumor tissue. Therefore, to further investigate the effect of VIS on cancer cell lines, we used a 3D cell culture model that simulates some aspects of intercellular interactions (Figure 19, Table 3). In spheroids formed from the sensitive cell line, we did not observe any significant effect of VIS on the response to TOP, and no significant changes in the IC_50_ value were noted. The determined IC_50_ for TOP alone was 8.38 ng/mL, compared to 7.78 ng/mL (10 µM), 8.05 ng/mL (25 µM), and 8.04 ng/mL (50 µM). In contrast, the addition of VIS into spheroids formed from TOP-resistant cell lines resulted in an increased sensitivity to TOP, and the effect was VIS concentration-dependent. Namely, in A2780TR1 cell line, co-treatment with 10 µM VIS resulted in a 5.23-fold decrease in IC_50_ value (IC_50_ = 361 ng/mL vs. IC_50_ = 68.9 ng/mL, *p* < 0.01), and co-treatment with VIS at concentration of 25 µM and 50 µM resulted in 8.79-fold (IC_50_ = 361 ng/mL vs. IC_50_ = 41 ng/mL, *p* < 0.01) and 8.78-fold (IC_50_ = 361 ng/mL vs. IC_50_ = 41,1 ng/mL, *p* < 0.001) decrease in the IC_50_ values, respectively. In the A2780TR2 cell line, co-treatment with 10 µM VIS resulted in a 3.25-fold decrease in IC_50_ value (IC_50_ = 319 ng/mL vs. IC_50_ = 98.1 ng/mL, *p* < 0.05). Co-treatment with VIS at concentrations of 25 µM and 50 µM resulted in 8.00-fold (IC_50_ = 319 ng/mL vs. IC_50_ = 39.8 ng/mL, *p* < 0.01) and 12.1-fold (IC_50_ = 319 ng/mL vs. IC_50_ = 26.3 ng/mL, *p* < 0.01) decrease in the IC_50_ values, respectively.

#### 2.2.8. Effect of VIS on BCRP Protein Activity in a 3D Model

**Figure 20:**
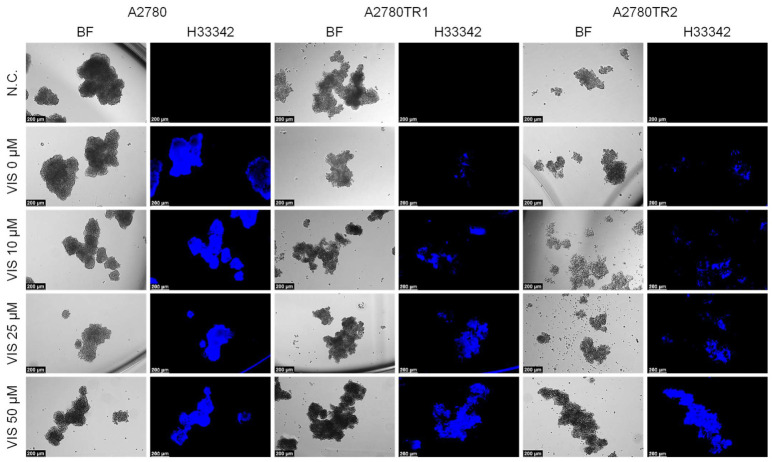
Fluorescent microscopy analysis of H33342 accumulation (blue) in 3D model in drug-sensitive A2780 cell line and TOP-resistant cell lines A2780TR1 and A2780TR2 in the absence or presence of Vismodegib (VIS) at concentrations of 10, 25, and 50 µM. Cells were pretreated with VIS for two hours, and then, fluorescence imaging and bright field visualization (BF) were performed. Scale bar = 200 µm.

**Figure 21:**
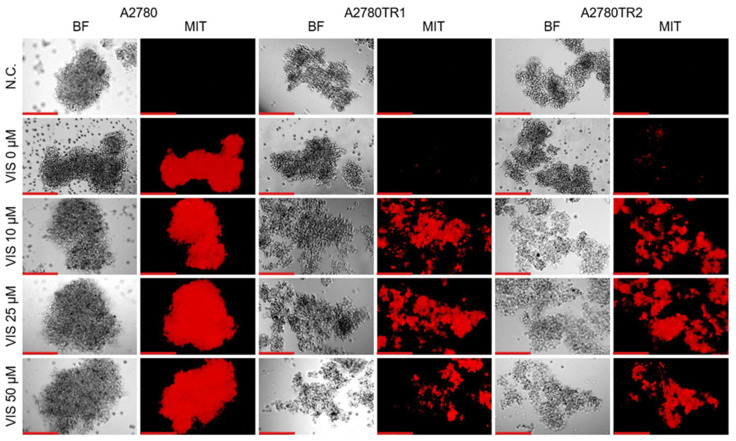
Fluorescent microscopy analysis of MIT accumulation (red) in 3D model in drug-sensitive A2780 cell line and TOP-resistant cell lines A2780TR1 and A2780TR2 in the absence or presence of Vismodegib (VIS) at concentrations of 10, 25, and 50 µM. Cells were pretreated with VIS for two hours, and then, fluorescence imaging and bright field visualization (BF) were performed. Scale bar = 200 µm.

As in the 2D model, we tested whether VIS affected BCRP protein activity in 3D spheroids. This was verified with fluorescence microscopy by examining the accumulation of H33342 and MIT. Similarly to the 2D cell culture, we observed high accumulation of H33342 and MIT days in 3D spheroids from A2780 cell lines, regardless of VIS concentration (Figure 20 and Figure 21). However, in spheroids from TOP-resistant lines, cultured without VIS, we did not observe accumulation of any substrate. Treatment with VIS resulted in a concentration-dependent increase in H33342 accumulation in both resistant cell lines (Figure 20). In the case of MIT, clear accumulation was observed in the presence of VIS. However, the effect seems not to be concentration-dependent (Figure 21).

#### 2.2.9. The Response of Drug-Sensitive and Resistant Cell Lines to VIS Treatment in the 3D Model

**Figure 22:**
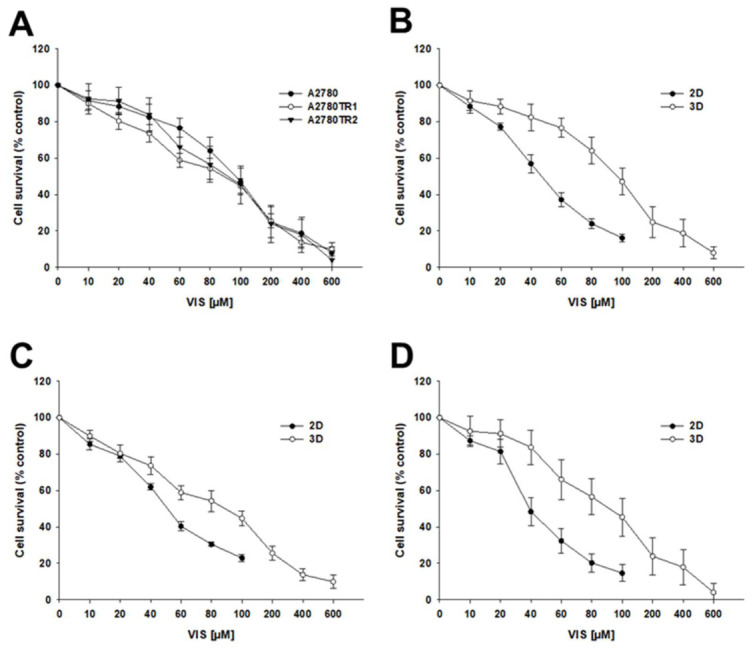
(**A**) Response of TOP-sensitive and resistant cell lines to VIS treatment in 3D cell culture conditions. Cell lines were seeded into 96-well plates. After spheroid formation, the cells were treated with increasing concentrations of VIS. Following 72 h-long incubation, the MTT cell survival assay was performed to determine cell viability. Comparison of the response to VIS in 2D and 3D conditions in A2780 (**B**), A2780TR1 (**C**), and A2780TR2 (**D**) cell lines. The cell viability assay was expressed as a percentage of the untreated control (mean ± SEM).

The effect of VIS on the tested cell lines was also investigated using a 3D model. We observed a very similar concentration-dependent response in all investigated cell lines (Figure 22A), which resulted in similar IC_25_ and IC_50_ values (Table 4). The determined IC_25_ of VIS for 3D culture of the A2780 cell line was 56.01 µM, and the IC_50_ was 107.14 µM. The IC_25_ for the A2780TR1 cell line was 32.42 µM, and the IC_50_ for this cell line was 85.01 µM. The determined IC_25_ value for the A2780TR2 cell line was 45.46 µM, and the IC_50_ was 106.07 µM.

Subsequently, we compared the response to VIS of cell lines grown as a 2D monolayer to the same cell lines grown as 3D spheroids. For all tested cell lines, the shape of the response curve in 3D cell culture conditions was similar to that in 2D conditions. However, the response curve was shifted to the right (Figure 22B–D), resulting in the higher IC_50_ values (Table 5). In the A2780 cell line in the three-dimensional model, the IC_50_ was 2.32-fold higher than in the 2D model (IC_50_ = 107.14 µM vs. IC_50_ = 46.13 µM, *p* < 0.05), (Figure 22B). In the A2780TR1 cell line, we observed 1.67-fold higher IC_50_ value in spheroids than in the monolayer (IC_50_ = 86.01 µM vs. IC_50_ = 51.46 µM, *p* < 0.05) (Figure 22C). In the A2780TR2 cell line, the IC_50_ was 2.49-fold higher in the 3D model (IC_50_ = 106.07 µM vs. IC_50_ = 42.54 µM) (Figure 22D).

#### 2.2.10. The Effect of VIS Treatment on Spheroid Structure and Viability

**Figure 23:**
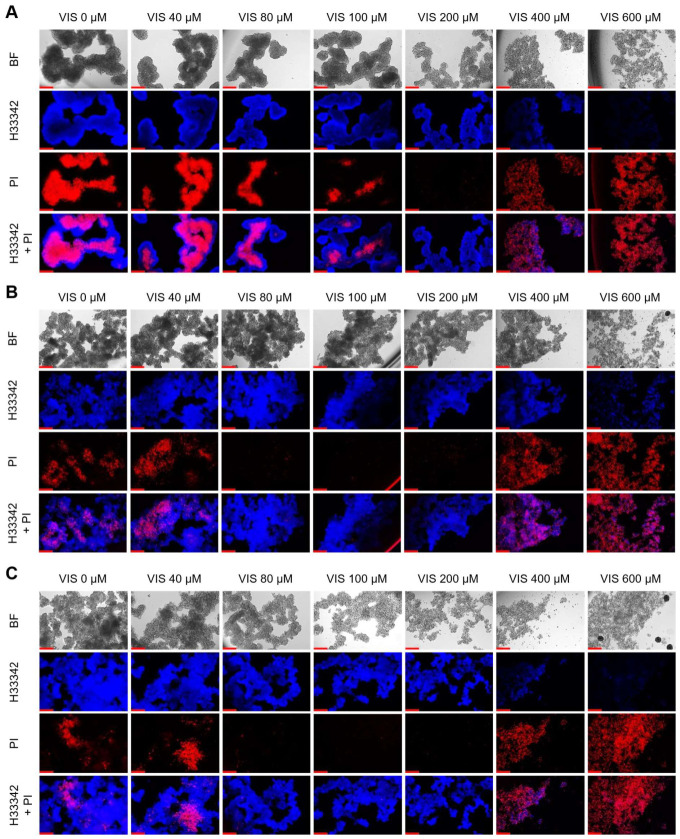
Fluorescent microscopy analysis of H33342 (blue) and PI (red) accumulation in 3D model in drug-sensitive A2780 cell line (**A**) and TOP-resistant cell lines A2780TR1 (**B**) and A2780TR2 (**C**) in the absence and presence of VIS at concentrations of 40, 80, 100, 200, 400, 600 µM. The cells were treated with VIS for 72 h, and then, a fluorescence analysis and bright field visualization (BF) were performed. Scale bar = 200 µm.

Next, we investigated the structure and viability of spheroids after 72 h of treatment with increasing VIS concentrations. In the control samples, we observed very dense spheroids in the A2780 cell line and dense spheroids in the A2780TR1 and A2780TR2 cell lines, with the presence of a necrotic core, especially visible in the A2780 cell line. Clear PI accumulation and no H33342 accumulation were observed in the necrotic core. An increase in VIS concentration resulted in the relaxation of spheroid structure, reflected by a decreased PI accumulation in the spheroid core. In the A2780 cell line with VIS at a concentration of 200 µM, and in A2780TR1 and A2780TR2 cell lines with VIS at concentrations of 80, 100, and 200 µM, we did not observe PI accumulation in the core of spheroids. Further increase in VIS concentration resulted in decreased H33342 accumulation with a very low signal at a VIS concentration of 600 µM. In contrast, a strong increase in PI accumulation in all of the cell lines was observed (Figure 23).

#### 2.2.11. Effect of VIS on Colony Formation, Proliferation, and Migration of Ovarian Cancer Cells

**Figure 24:**
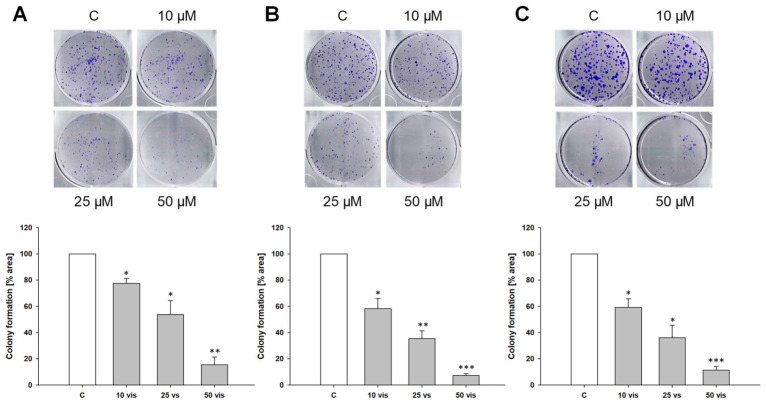
Colony formation assay in A2780 (**A**), A2780TR1 (**B**), and A2780TR1 (**C**) cell lines. The cell lines were cultured for 7 days with the appropriate concentrations of VIS, and then fixed and stained with crystal violet (visualization). The percentage of the area of colonies formed (chart) was calculated. For each cell line, the colony formation of the untreated control was defined as 100% (white bar), and the colony areas of samples treated with increasing concentrations of the inhibitor were expressed as percentages relative to the control (gray bars). The values were considered significant at * *p* < 0.05, ** *p* < 0.01 and *** *p* < 0.001.

To further investigate the effect of VIS on the A2780 and TOP-resistant cell lines, a colony formation assay was performed (Figure 24). Treatment with VIS in all tested cell lines resulted in a decrease in the area of colonies formed, and the effect was VIS concentration-dependent. Treatment with VIS at a concentration of 10 µM resulted in a decrease in the area of colonies to 80% of the control for the A2780 cell line and 60% for the A2780TR1 and A2780TR2 cell lines, *p* < 0.05. VIS at a concentration of 25 µM reduced the colony area to approximately 60% for the A2780 cell line, *p* < 0.05, and approximately 40% for the TOP-resistant cell lines, *p* < 0.01 and *p* < 0.05, respectively. VIS at a concentration of 50 µM decreased the colony area to approximately 20% (A2780), *p* < 0.01, and 15% (A2780TR1, A2780TR2), *p* < 0.001.

**Figure 25:**
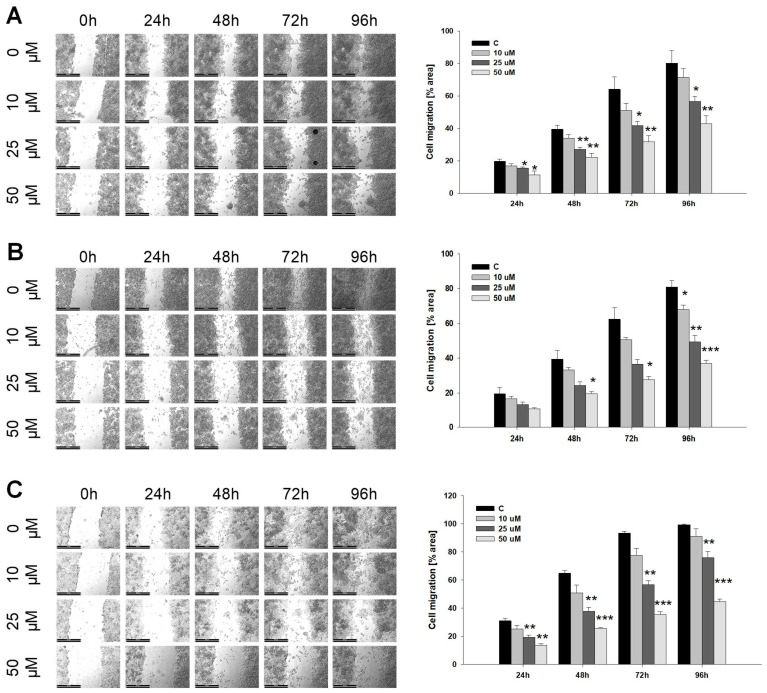
Wound healing assay with A2780 (**A**), A2780TR1 (**B**), and A2780TR2 (**C**) cell lines. Cell lines were cultured for 24 h with the appropriate concentrations of VIS, a wound was made, and cell migration was observed for 96 h, every 24 h. Additionally, the percentage of migration area (chart) was calculated. The values were considered significant at * *p* < 0.05, ** *p* < 0.01 and *** *p* < 0.001. Scale bar = 500 µm.

The effect of VIS on cancer cell migration was also investigated (Figure 25). In A2780 and A2780TR2 cell lines, we observed a statistically significant decrease in cell migration at VIS concentrations of 25 and 50 µM in all time points. In the A2780TR1 cell line, the inhibitory effect was observed at 50 µM, at time points of 48, 72, and 96 h, and at 10 and 25 µM after 96 h of treatment.

**Figure 26:**
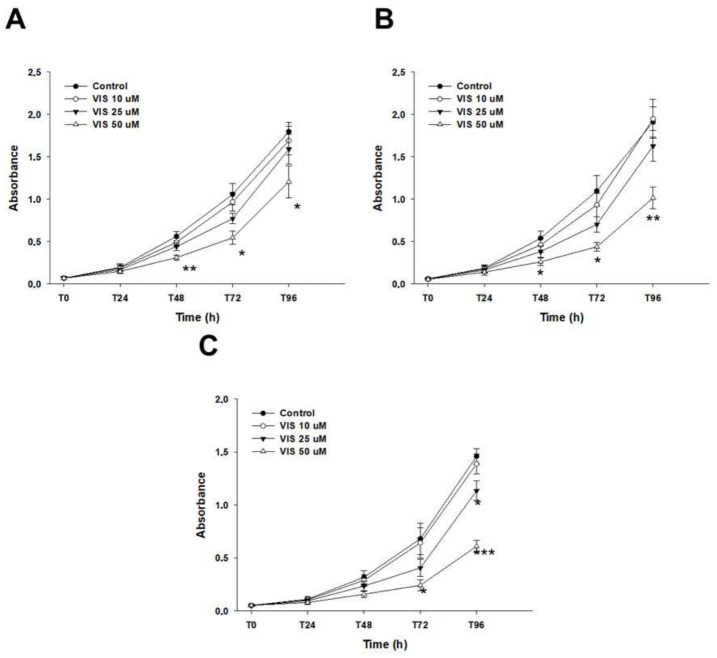
Analysis of cell proliferation after VIS treatment. Cell lines: A2780 (**A**), A2780TR1 (**B**), and A2780TR2 (**C**). Proliferation was checked every 24 h via the MTT assay. The proliferation rate of each line decreased after the highest dose of VIS was used. The values were considered significant at * *p* < 0.05, ** *p* < 0.01 and *** *p* < 0.001.

The effect of VIS on the proliferation of ovarian cancer cells was also tested (Figure 26). A 50 µM concentration of the inhibitor was shown to affect the proliferation of all tested cell lines. A reduction in proliferation rate was observed for the A2780 and A2780TR1 cell lines after 48 h and continued at subsequent time points. For the A2780TR2 cell line, a reduction in proliferation rate was observed after 72 h and continued at subsequent time points. Additionally, we observed a statistically significant decrease in A2780TR2 cell proliferation at a VIS concentration of 25 µM and after 96 h. TOP-resistant cell lines showed a greater decrease in proliferation after 96 h compared to the A2780 line. No effect on the proliferation of the tested cell lines was observed after treatment with 10 µM and 25 µM Vismodegib.

## 3. Discussion

Most cases of patients with ovarian cancer are sensitive to chemotherapy at the beginning of treatment. However, the development of drug resistance during treatment eventually leads to low effectiveness of chemotherapy [1]. Although different mechanisms of drug resistance can be developed in cancer, the acquired drug resistance usually presents as multiple drug resistance (MDR)—the resistance to most of the clinically used cytotoxic drugs [7,8]. Thus, scientists and clinicians try to find new therapeutic targets and novel anticancer drugs to increase the effectiveness of treatments. As the activity of CSCs’ signaling pathways is increased not only in CSCs themselves but also in other drug-resistant cells, CSCs’ signaling pathways seem to be an attractive therapeutic target in cancers [52]. One of the CSCs signaling pathways involved in the development of ovarian cancer is Sonic Hedgehog [33,34].

Thus, in this study, we used a Shh pathway inhibitor—Vismodegib (VIS) [35,36,37,38]—a drug approved to treat cancers [39,40,41,42], to check its effectiveness in drug-sensitive A2780 and TOP-resistant—A2780TR1 and A2780TR2—ovarian cancer cell lines [51].

Before starting the experiments with VIS, we shortly characterized the cell lines used in our study. As TOP resistance is often associated with BCRP drug transporter overexpression [11,15], we checked the expression of the BCRP gene and protein in the investigated cell lines. We observed high BCRP overexpression in both TOP-resistant cell lines with cell membrane localization, which is consistent with our previous studies [51,53]. As ALDH1A1 is the most popular marker of CSCs in solid tumors [24], we checked its expression in the investigated cell lines. Low increase in *ALDH1A1* transcript in both TOP-resistant cell lines correlated with the presence of ALDH1A1 protein only in a small population of the drug-resistant cells. Similarly, the presence of ALDH1A1^+^ and ALDH1A1^-^ cells in drug-resistant ovarian cancer cell lines was previously observed by our team and by others [54,55,56,57]. As drug resistance is often associated with increased protein phosphorylation [58,59] and decreased activity of the phosphatases [60], we compared PTPRK and pTYR levels in the investigated cell lines. The PTPRK expression was decreased in drug-resistant cell lines, while the pTYR level was increased in drug-resistant cell lines, which was consistent with our previous findings [10].

Then, we compared the expression of the most important genes involved in the Shh pathway [27,28]. Since very similar levels of *SMO*, *PCTCH1*, *SHH*, *ILK*, and *GLI1* genes were observed in both sensitive and TOP-resistant cell lines, we concluded that the expression of these genes does not seem to be related to TOP resistance. Downregulation of *GLI2* and upregulation of *GLI3* genes in the A2780TR1 cell line can suggest some relation to drug resistance, although the expression level of *GLI2* and especially *GLI3* genes was very low in comparison to *GLI1* gene, suggesting that *GLI1* transcription level changes play the most important role in these cell lines [61].

After characterizing the investigated cell lines, we checked the cytotoxic effect of VIS on A2780, A2780TR1, and A2780TR2 cell lines. In all of the cell lines, we observed very similar, concentration-dependent response curves and similar IC_25_ and IC_50_ values. As the SHH pathway is a target of VIS, similar response curves suggest similar activity of this pathway in both drug-sensitive and resistant cell lines. A similar response curve and IC_50_ values for VIS were observed in meduloblastoma cell lines, with IC_50_ values between 52 and 84 µM for VIS [62]. The inhibition of breast cancer cell lines’ survival by VIS was observed at concentrations of 3, 6, and 12 µM [63]. In pancreatic cancer cell lines, the reduction of cell viability to 50% after 72 h was observed at concentrations between 5 and 10 µM [64]. In two lung cancer cell lines, HCC and H1339, the IC_50_ for VIS after 72 h of exposure was about 50 µM [65]. In neuroblastoma cell lines (SH-SY-5Y, IMR-32, and SK-N-BE(2)), the response curves to VIS were very similar to ours, with determined IC_50_ values equal to 77, 61, and 76 µM [66]. Thus, IC_50_ values observed in our cell lines seem to be similar to those observed in other studies and are in the micromolar range of concentrations.

Next, we studied the mechanisms of cell death following VIS treatment. In all of the cell lines, we observed a concentration-dependent increase in the number of necrotic cells, while the number of early and late apoptotic cells did not change significantly. This indicates that VIS causes cell death mainly by inducing necrosis. This is in contrast to the study of Wu et al., who observed that VIS concentration causes a time-dependent increase in the percentage of apoptotic cells in colon cancer cell lines [67]. In neuroblastoma cell lines, VIS induced mainly late apoptosis [66]. In oral squamous carcinoma cells, VIS induced cell death by induction of apoptosis after 72 h of treatment [68]. In the gastric cancer cell line SGC-7901, lower VIS concentrations (5, 10, and 20 µM) induced mainly apoptotic cell death, and a higher concentration (50 µM) induced necrotic cell death as well [69]. Thus, the kind of cell death induced by VIS seems to depend on the kind of cells experimented on.

As the pTyr level can be associated with drug resistance [58,59], we also checked the effect of VIS on pTyr. At a VIS concentration of 50 µM, we observed decreased pTYR level, especially in the A2780 cell line. However, at a lower VIS concentration, we observed a similar level of total pTYR, which suggests the changes in signal transduction intensity are not the main mechanisms of VIS action in the investigated cell lines.

Knowing that VIS shows cytotoxic effect against both sensitive and resistant cell lines, in the next part of our study, we wanted to establish whether VIS increases sensitivity to cytotoxic drugs. Thus, we investigated the effect of VIS in concentrations corresponding to IC_10_ and IC_25_ on the cytotoxic effect of TOP in the investigated cell lines grown as a monolayer. In the A2780 cell line, we observed very similar response curves to TOP in the presence and absence of VIS, resulting in a similar IC_50_ value. In contrast, in both TOP-resistant cell lines, co-treatment with VIS produced a response curve with the same shape as that observed for TOP alone. However, in the presence of VIS, the curves were shifted to the left, resulting in decreased IC_50_ values for TOP. We previously observed a similar effect with the BCRP protein inhibitor elacridar [70]. The differences in VIS effect on TOP resistance between the drug-sensitive A2780 cell line and the TOP-resistant cell lines suggest that low-concentration VIS treatment causes changes in BCRP protein expression and/or activity, rather than other cellular processes. Thus, in the next part of our study, we wanted to check both possibilities.

In the literature, it was described that SMO protein antagonist, like cyclopamine or LDE225, used on PAC-resistant cell lines caused PAC-sensitization and decreased expression of drug transporters like P-gp [71]. In another study, treatment with cyclopamine of SEG-1 (esophageal adenocarcinoma), LNCaP (prostate carcinoma), PC3 (prostate carcinoma), and DM14 (metastatic squamous cell carcinoma) cell lines also resulted in downregulation of P-gp and BCRP expression and increased accumulation of cytotoxic drugs in cancer cells [30]. Thus, we wanted to check the effect of VIS on BCRP protein expression. Using WB, we did not observe any effect of VIS on BCRP protein expression after 72 h of treatment, even at a concentration of 50 µM. Next, we performed immunofluorescence and flow cytometry experiments after 72 h of VIS treatment to check the BCRP protein activity. However, VIS was washed from the cell culture medium before measurement of BCRP transport activity, to exclude direct effect on BCRP transporter activity. Results of both experiments suggest that long-time VIS treatment does not change BCRP activity/expression, as BCRP substrate accumulation did not increase in TOP-resistant (BCRP-expressing) cells treated with VIS. We also looked through the PubMed database. However, we did not find papers describing changes in BCRP expression after VIS treatment.

Another possibility is that VIS is directly involved in the BCRP protein transporter activity. For experiments to confirm that, the cells were pretreated with VIS for one hour, and VIS was present in the medium during fluorescence experiments. Both IF and FC experiments using H33342 and MIT as BCRP substrates showed that VIS treatment results in higher accumulation of H33342 and MIT in both TOP-resistant cell lines to a level similar to that of the TOP-sensitive cell line. Furthermore, FC analysis that is more accurate showed that the effect of VIS is concentration-dependent. These results clearly correlate with the results of MTT co-treatment experiments. We observed similar results before, when using a known P-gp and BCRP inhibitor, elacridar [70,72,73]. Thus, our results suggest that VIS increases the cytotoxic effect of TOP, mainly by inhibition of BCRP transporter activity. Looking through the literature data, we found only one paper describing the effect of VIS on BCRP protein activity and reversion of the MDR [48]. Firstly, the authors who used HEK293 with genetically engineered BCRP overexpression showed that VIS inhibits BCRP protein activity and increases retention of BCRP fluorescent substrate BODIPY-prazosin. Next, using non-small cell lung carcinoma cells NCI-H460/par and NCI-H460/MX20, which overexpress ABCG2, they showed that co-treatment with VIS re-sensitizes these cell lines to MIT and TOP. Thus, they observed a similar effect to that observed in our study. A strong increase in TOP cytotoxic effect after co-treatment with VIS was observed in three neuroblastoma cell lines: SH-SY-5Y, IMR-32, and SK-N-BE(2) [66]. All these cell lines express BCRP protein, especially SK-N-BE(2), which corresponds to a higher TOP IC_50_ value in the SK-N-BE(2) cell line [66]. Thus, it is highly probable that a strong synergistic cytotoxic effect of TOP and VIS resulted from BCRP inhibition. Unfortunately, authors did not investigate the effect of VIS on BCRP activity [66].

Although a 2D monolayer is a useful model for studying drug resistance at the cellular level, a cancer tumor is a complex 3D structure characterized by dense cellular architecture, the presence of ECM molecules, and CAM-DR, which limit drug diffusion and increase resistance to therapy [16,17,18,19,20]. Thus, we wanted to check the effect of VIS on the investigated cell lines in a 3D spheroid model that is more similar to a real tumor structure. Previously, we compared BCRP protein expression in investigated cell lines grown as 2D monolayers and 3D spheroids. Our results showed very similar BCRP protein expression levels in both models [53,70]. In the 3D model, all cell lines were also more resistant to TOP than in the 2D model—this is consistent with our previous study [53].

Co-treatment experiments with VIS and TOP in 3D spheroids showed very similar results to those in the 2D model. We did not observe any effect of VIS on the level of TOP resistance in the A2780 cell line. In contrast, VIS concentration-dependent effect on TOP resistance was observed in both TOP-resistant cell lines, suggesting a mechanism related to inhibition of BCRP protein activity. Thus, similarly to the experiments performed in the 2D model, we investigated the effect of VIS on the accumulation of BCRP substrates H33342 and MIT in 3D spheroids. Strong accumulation of both substrates was observed in the A2780 cell line, independent of VIS presence, suggesting good diffusion properties of both dyes through the spheroid structure. However, no substrate accumulation in the absence of VIS was observed in spheroids of both TOP-resistant cell lines, indicating activity of the BCRP protein. Treatment with VIS resulted in strong accumulation of H33342 or MIT in TOP-resistant spheroids, suggesting inhibition of BCRP protein activity. These results clearly correlate with MTT results. We did not find similar studies in the literature, so we cannot directly compare our data with the results of others. However, we have previously investigated the effect of the known BCRP inhibitor elacridar on BCRP activity in spheroids, showing a very similar effect to VIS. Thus, the results acquired with elacridar seem to support our hypothesis that VIS is also capable of BCRP inhibition in 3D spheroids. The similarity of observed effects suggests that VIS may act through a comparable functional mechanism, namely the inhibition of BCRP transport activity. Importantly, the consistency between increased substrate accumulation and reduced cell viability (MTT assay) further supports this hypothesis. Given that BCRP plays a critical role in drug resistance in 3D tumor models, the ability of VIS to reproduce the effects of elacridar highlights its potential as a modulator of multidrug resistance in spheroids. In summary, 2D and 3D co-treatment results suggest that VIS is a promising candidate for improving the efficiency of TOP and probably other drugs in cancer cells. Our data seems to be supported by others. In a study with neuroblastoma cell lines, VIS combined with TOP caused a significant reduction in size and total number of neurospheres, compared to the efficiency of these agents used separately [66]. Also, in a xenograft study, VIS combined with TOP significantly reduced the tumor growth compared to the control, resulting in a significantly higher survival of mice [66].

Next, we wanted to compare the effect of VIS in drug-sensitive and drug-resistant cell lines in 2D and 3D cell culture conditions. The response curves to VIS in spheroids from both TOP-sensitive and resistant cell lines are very similar, resulting in comparable IC_25_ and IC_50_ values. Moreover, the comparison of responses to VIS in the same cell lines grown in 2D and 3D cell culture conditions shows a very similar shape of the response curve. However, in the 3D conditions, the curve is shifted to the right, resulting in increased IC_50_ values. The increase in IC_50_ value is not very significant, only about 2-fold, which suggests that VIS migrates easily through dense spheroids’ structure. Effective VIS diffusion through a spheroid structure is a very desirable feature of anticancer drugs. Many clinically used anticancer drugs, such as PAC, show limited diffusion potential [23,24], causing a very high increase in resistance level between 2D monolayer and 3D spheroids, as we observed previously [19,51]. In contrast, in the case of TOP, we noticed a low to moderate increase in resistance between the 2D and 3D models [19,51]. The insignificant difference in IC_50_ value for VIS between 2D and 3D models suggests that VIS is a promising candidate to treat ovarian cancer, both alone or in combination with TOP.

Afterward, we researched the mechanism of cell death in spheroids after VIS treatment. To do that, we compared responses to VIS in all three cell lines growing in 3D cell culture conditions using PI/H3342 staining. PI labels only dead cells, as it is not capable to diffuse trough live cell membranes; thus, it labels mainly necrotic cells [74]. In contrast, H33342 easily migrates through the membrane, binds to DNA and labels live cells. In apoptotic cells, the H33342 signal increases because of the higher chromatin condensation. In contrast, necrotic cells usually present low H33342 (blue) staining. Thus, the necrotic cells usually show low to average blue (H33342) and strong red (PI) fluorescence. The A2780 cell line formed high-density spheroids, while the density of A2780TR1 and A2780TR2 spheroids was medium. This seems to be reflected by the presence of “natural” necrotic core in denser spheroids [75], which was stained by PI, but remained free of H33342 dye. The presence of a natural necrotic core results from the size and/or high cell density that limits the diffusion of nutrients and gases [75,76]. The necrotic core was clearly visible in dense A2780 spheroids. Treatment with VIS in all cell lines resulted in a concentration-dependent relaxation of spheroid density, reflected by the loss of necrotic core, indicated by loss of PI staining, presence of H33342 staining, and is also visible in bright field (BF) observation. In VIS at concentrations of 400 and 600 µM, we observed a further change in spheroid structure in BF, a concentration-dependent increase in PI staining, and a decrease in H33342 staining, indicating necrosis. Necrotic cell death following VIS treatment correlates with 2D flow cytometry results. This can be important from the therapeutic point of view. Many chemotherapeutic agents fail because tumor cells manage to evade apoptosis. Activation of necrosis by VIS in our cell lines has a therapeutic advantage of bypassing the apoptotic blocks typical of Topotecan-resistant cells [77].

Next, we tested the effect of VIS at concentrations of IC_10_ (10 µM), IC_25_ (25 µM) and IC_50_ (50 µM) on other cellular processes related to cancer progression. In all of the cell lines, we observed a concentration-dependent reduction of colony formation after long-term VIS treatment, indicating that VIS effectively eliminates cancer cells. Very similar results were observed in glioma cell lines U251, C6, and A172, where VIS in the same concentration effectively inhibited the colony formation [78]. Similar concentrations (20 and 40 µM) of VIS inhibited colony formation in head and neck squamous cell carcinoma (HNSCC) [79]. Inhibition of colony formation by VIS suggests that it effectively inhibits cancer progression. In a simple wound healing assay, we observed a concentration-dependent inhibition of cell migration with some differences between cell lines. In general, VIS treatment effectively inhibited cell migration at concentrations close to IC_25_ and IC_50_, suggesting that VIS exhibits inhibitory potential toward cancer cell migration and metastasis. Similar observations were made by others. At the same concentrations, VIS inhibited the migration of glioma cell lines U251, C6, and A172 [78]. Inhibitory effect of VIS at a concentration of 10 µM was also observed in MCF10DCIS.com [80] and SUM159 [81] breast cancer cell lines. In contrast to a strong inhibitory effect on colony formation and medium effect on cell migration, an impact on cell proliferation was rather moderate, as we only observed the inhibition of cell proliferation in IC_50_ concentration, following long-term exposure. A similar concentration of VIS was used in studies conducted on the SGC-7901 gastric cancer cell line [69] and in colon cancer cell lines Caco-2 and Ht-29, showing a concentration-dependent inhibitory effect [67]. Summarizing this part of the study, VIS effectively inhibits processes related to cancer progression.

In conclusion, we investigated the effect of Vismodegib, a Shh pathway inhibitor, in drug-sensitive and resistant ovarian cancer cell lines. Using both 2D and 3D cell culture models, we observed that VIS shows a similar cytotoxic effect against both drug-sensitive and resistant cell lines. The main mechanism of cell death in both cell culture conditions seems to be related to cell necrosis. Low difference in IC_50_ values between 2D and 3D cell culture conditions suggests good diffusion properties of VIS through the dense intercellular structure, which is a very desirable feature of anticancer drugs. Apart from acting in monotherapy, VIS very effectively increases the efficiency of TOP by inhibition of BCRP protein activity. An increase in chemotherapy efficiency is also a very desirable feature of new anticancer drugs. Finally, VIS effectively inhibits processes related to cancer progression, like cell migration and colony formation.

In summary, our results suggest that VIS is a promising candidate as an anticancer drug in ovarian cancer; however, continued experiments are necessary to fully determine the usefulness of this inhibitor in ovarian cancer treatment. The main limitation of this study is that all experiments were conducted using in vitro models. Confirmation of these results in more clinically relevant models, including the CAM (chorioallantoic membrane) model and mouse xenograft models, remains necessary.

## 4. Materials and Methods

### 4.1. Reagents and Antibodies

The cell culture reagents, including fetal bovine serum (FBS), MEM medium, penicillin, streptomycin, L-glutamine, amphotericin B (25 μg/mL), and trypsin-EDTA solution, were purchased from Sigma (St. Louis, MO, USA). DPBS was acquired from Corning (Corning, NY, USA). Vismodegib (VIS) was purchased from Molnova (Ann Arbor, MI, USA). Topotecan (TOP) and mitoxantrone (MIT) were obtained from Selleckchem (Houston, TX, USA). Cell proliferation Kit I (MTT)—Thiazolyl Blue Tetrazolium Bromide and bisBenzimide H33342 trihydrochloride (H33342) were purchased from Sigma (St. Louis, MO, USA). Propidium iodide (PI) was purchased from Thermo Fisher (P3566, Waltham, MA, USA). Annexin V was obtained from Thermo Fisher (a35110, Waltham, MA, USA).

qPCR reagents, including M-MLV reverse transcriptase kit (28025013) and RnaseOUT (10777019), were purchased from Invitrogen by Thermo Fisher (Waltham, MA, USA). All Western blot reagents (including gels, protein markers, and membranes) were acquired from Bio-Rad (Bio-Rad Laboratories, Hemel Hempstead, UK).

Rabbit anti-BCRP/ABCG2 antibody (D5V2K) was obtained from Cell Signaling (Danvers, MA, USA). Rabbit anti-ALDH1A1 was acquired from Abcam (ab52492, Cambridge, UK). Mouse anti-PTPRK and mouse anti-pTYR were obtained from Santa Cruz Biotechnology (Dallas, TX, USA). The secondary antibodies of HRP-conjugated Goat Anti-Rabbit IgG (SA00001-2), goat anti-mouse HRP-conjugated (SA00001-1-A), anti-β-actin (66009-1-Ig), and anti-GAPDH (60004-1-Ig) were purchased from Proteintech (Rosemont, IL, USA). Alexa Fluor^®^ 488 AffiniPure™ Donkey Anti-Rabbit IgG (H + L) (711545-152) and Alexa Fluor^®^ 488 AffiniPure™ Donkey Anti-Mouse IgG (H + L) (715-545-150) were acquired from Jackson ImmunoResearch Laboratories (Ely, Cambridgeshire, UK).

### 4.2. Cell Culture

The human ovarian carcinoma cell line A2780 was obtained from American Type Culture Collection (ATCC, Manassas, VA, USA). TOP-resistant lines A2780TR1 and A2780TR2 were obtained by sequentially exposing A2780 cells to increasing concentrations of TOP. The final concentration used for selecting the resistant cells was 24 ng/mL of TOP.

All cell lines were cultured in MEM medium with 10% (*v*/*v*) fetal bovine serum content and 2 mM L-glutamine, as well as the antibiotics streptomycin (100 µg/mL), penicillin (100 I.U./mL), and amphotericin (0.25 µg/mL), under typical culture conditions (37 °C, 5% CO_2_). For TOP-resistant lines, the cells were cultured with TOP at a final concentration of 24 ng/mL.

### 4.3. RNA Isolation, cDNA Synthesis, and QPCR

RNA was isolated using the Gene Matrix Universal RNA Purification Kit (EURx, Gdańsk, Poland), according to the manufacturer’s instructions. The RNA concentration was determined by measuring the absorbance at 260 and 280 nm. Then, cDNA synthesis was performed using the CFX Opus 96 Real-Time PCR system (Bio-Rad Laboratories, Hemel Hempstead, UK). Each reaction consisted of 1.5 µg of RNA, 1 µL of oligodT18A primer (IBB PAN, Warsaw, Poland), and 1 µL of dNTP mix (Thermo Fisher, Waltham, MA, USA, R0192). Samples were denatured at 65 °C for 5 min, after which 4 µL of 5× First Strand buffer, 2 µL of DTT, 0.5 µL of RNase inhibitor (RnaseOUT), and 0.5 µL of reverse transcriptase (M-MLV RT) were added. The reaction was carried out for 60 min at 37 °C, followed by 15 min at 75 °C.

A real-time quantitative PCR (real-time PCR) reaction mixture for a single sample was prepared by combining 12.5 μL of Takyon™ ROX SYBR^®^ MasterMix blue dTTP (Eurogentec, Searing, Belgium), 1 μL of each sequence-specific primer (7.5 μM) provided by Oligo.pl (Warsaw, Poland) (Table 6), 9.5 μL of nuclease-free water, and 1 μL of cDNA solution. GADPH was used as the reference gene, and nuclease-free water was used as the negative control. Amplification was performed using a CFX Opus 96 Real-Time PCR system (Bio-Rad Laboratories, Hemel Hempstead, UK) according to the following thermal cycler program: initial denaturation at 95 °C for 15 min, followed by 45 cycles of denaturation at 95 °C for 15 s, annealing at 60 °C for 30 s, and extension at 72 °C for 30 s; final extension was performed at 72 °C for 30 s.

### 4.4. Protein Isolation and Western Blot Analysis

The cell lines were seeded into 6-well plates at 2 × 10^5^ cells per well in a volume of 2000 µL of culture medium. After 48 h of culture, the cell medium was replaced. Depending on the experiment, the medium was either without an inhibitor or with VIS at concentrations of 10, 25, or 50 µM. After 72 h, cellular protein was isolated. The cells were washed with Phosphate-Buffered Saline (PBS) with Ca^++^/Mg^++^ ions three times and were lysed using RIPA buffer containing protease inhibitor cocktail and PhosSTOP phosphatase inhibitor cocktail (Roche Diagnostics GmbH, Mannheim, Germany) for 80 min at 4 °C. Then, the cells were centrifuged at 13.4 × 10^3^ rpm for 30 min at 4 °C. Protein concentrations were determined using the Bradford protein assay system (Bio-Rad Laboratories, Hemel Hempstead, UK). Protein samples were prepared from 7 µg protein, 4× loading buffer (Bio-Rad Laboratories, Hemel Hempstead, UK), and 10% β-mercaptoethanol and separated on a 4–20% mini-PROTEAN^®^ TGX™ gel (Bio-Rad Laboratories, Hemel Hempstead, UK) using SDS-PAGE electrophoresis conducted with Mini-PROTEAN Tetra Vertical Electrophoresis Cell with PowerPac™ Basic Power Supply (Bio-Rad Laboratories, Hemel Hempstead, UK). Proteins were then transferred to a nitrocellulose membrane (except for pTYR protein in experiment without the inhibitor, where a PVDF membrane was used instead), with the Trans-Blot^®^™ transfer system (Bio-Rad Laboratories, Hemel Hempstead, UK), blocked with BSA 5% (for pTYR) or 5% skimmed milk powder solution in TBS/Tween (0.1 M Tris-HCl, 0.15 M NaCl, 0.1% Tween 20) for the other proteins, for 1 h. Incubation with primary antibodies, such as anti-BCRP (1:2000), diluted in 5% skimmed milk solution, or anti-pTYR (1:1000), diluted in 5% BSA solution, was performed overnight, followed by a 3 h incubation with HPR-labeled goat secondary antibody (1:10,000). Then, the signals were developed using a chemiluminescence detection system (ECL, Femto Super Signal Reagent (Bio-Rad Laboratories, Hemel Hempstead, UK)) and ChemiDoc™ (Bio-Rad Laboratories, Hemel Hempstead, UK). Following, the membranes were washed with TBS/Tween (0.1 M Tris-HCl, 0.15 M NaCl, 0.1% Tween 20) and incubated with antibodies directed against β-actin (1:7500) or against GADPH (1:40,000), followed by a 3 h incubation with HPR-labeled goat secondary antibody (1:10,000). Signals were then again developed using a chemiluminescence detection system (ECL, Femto Super Signal Reagent) and ChemiDoc™ (Bio-Rad Laboratories, Hemel Hempstead, UK). β-Actin or GADPH was used as the reference protein.

### 4.5. Immunofluorescence

The cells were cultured on glass slides placed in the wells of a 24-well culture plate until approximately 70% confluence was reached. The medium was then removed, and the cells were washed with PBS, and were fixed and permeabilized with ice-cold acetone/methanol (1:1) for 15 min at room temperature (RT). After further washes in PBS, cells were blocked in 3% BSA for 30 min (RT) and then incubated with a primary antibody (anti-BCRP (1:300); anti-ALDH1A1 (1:200); anti-PTPRK (1:100) or anti-pTYR (1:100)) for 2 h at room temperature. After washing three times with PBS, the cells were incubated with Alexa Fluor^®^488-conjugated secondary antibody (West Grove, PA, USA) (goat anti-rabbit antibody (1:200) or goat anti-mouse antibody (1:200)) for 1 h (RT). Slides were then washed three times with PBS, once with distilled water, and mounted in mounting medium containing DAPI (Sigma, F6057) to visualize cell nuclei. Protein expression was analyzed by fluorescence microscopy (Zeiss Axio-Imager.Z1, Oberkochen, Germany) using Zen Blue v3.3 software. Observations were performed using a 40× objective.

### 4.6. MTT Assay in 2D Cell Culture Conditions

Cell lines were seeded in 96-well plates at a density of 3 × 10^3^ cells per well in a volume of 200 µL of culture medium. After 48 h of culture, the cells were treated with VIS (10, 20, 40, 60, 80, 100 µM). After 72 h, the medium containing the inhibitor was removed, and a mixture of 100 µg of MTT in 170 µL of medium (final MTT concentration, 0.59 µg/mL) was added to each well. The one-hour incubation was terminated by replacing the MTT-medium mixture with 200 µL of DMSO to dissolve the formazan crystals. The absorbance of the resulting mixture was measured at 570 nm and 720 nm using a Synergy LX Multi-Mode Reader (BioTek Instruments, Inc., Winooski, VT, USA). Each experiment was performed in duplicate and repeated at least four times in independent series. Based on the obtained results, the IC_50_ value was determined.

Cell viability was then assessed during 72 h of TOP and VIS co-treatment. The experiment was performed as above, with a change in the composition of the experimental medium. Namely, this medium contained 0, 10 µM, or 25 µM VIS and increasing concentrations of TOP. The obtained results allowed for the determination of the IC_50_ for TOP in the presence and absence of the inhibitor.

### 4.7. MTT Assay in 3D Cell Culture Conditions

The cells were seeded into non-adherent 96-well plates (BRAND plates inter Grade, F-bottom, 781902, Merck, Darmstadt, Germany) at 1 × 10^4^ cells per well. After 48 h, spheroids formed, and 100 µL of medium was replaced with a mixture of VIS (in the final concentrations of 0, 10, 25, or 50 µM) and appropriate concentrations of TOP. Under these conditions, the spheroids were cultured for 72 h. Cell viability was then determined using the Cell Proliferation Kit I (Roche, Basel, Switzerland, cat. no. 11465007001). Specifically, 100 µL of medium was removed from the wells, and 10 µL of MTT reagent (to achieve the final concentration of 0.45 µg/mL) was added. After 4 h, 100 µL of solubilization buffer was added, and the plates were left at 37 °C overnight. Absorption was measured using a Synergy LX Multi-Mode Reader (BioTek Instruments, Inc., Winooski, VT, USA) at wavelengths of 570 nm and 720 nm. Each experiment was performed in duplicate and repeated at least four times. The IC_50_ was then determined.

Additionally, the IC_25_ and IC_50_ were determined for VIS alone, with spheroids cultured with various concentrations of VIS. The MTT assay was performed as described above.

### 4.8. Apoptosis/Necrosis Assay with Annexin V

VIS-treated tumor cells were analyzed by annexin V/PI double staining. Cancer cells were seeded at 100,000 cells per well on a 6-well plate. The next day, we added VIS at the following concentrations (0, 10, 25, and 50 µM) to the cells. After 72 h, the cells were trypsinized, centrifuged at 200× *g* for 5 min at room temperature, washed with 2 mL of PBS, and centrifuged under the same conditions. The cell pellet was suspended in 400 ul of binding buffer (ABB; 10 mM HEPES, pH 7.4, 140 mM NaCl, 2.5 mM CaCl_2_). Next, starting with 170 µL of cell suspension, we added 20 µL of 1 mg/mL PI, 2 µL of annexin, 8 µL of ABB buffer (achieving the final volume of 200 µL), and incubated for 20 min in the dark. Cells were washed twice with ABB buffer and resuspended in 400 µL of this buffer. Cells were analyzed on a MACSQuant 10 flow cytometer (Miltenyi Biotec, Bergisch Gladbach, Germany). The percentages of early apoptotic cells (annexin V positive, PI negative), late apoptotic cells (PI positive, annexin V positive), and necrotic cells (PI positive, annexin V negative) were then calculated using FACS-FlowJo 10.9 software.

### 4.9. Live-Cell Fluorescence (H33342 and MIT Accumulation) (2D)

#### 4.9.1. Long-Term VIS Incubation

The effect of VIS on the activity and synthesis of the transport protein BCRP was investigated by determining the change in dye accumulation in cell lines. The cell lines were seeded at a concentration of 1 × 10^4^ cells per well in a 24-well plate. After 48 h, the medium was replaced with 10 µM, 25 µM, or 50 µM VIS or medium without VIS. After 72 h of culture, VIS was washed from the cells using cell culture medium, and the cells were incubated for 1 h with H33342 (0.5 µg/mL) or MIT (0.2 µg/mL). Next, the cells were washed three times with cold PBS with 50 µM verapamil. Observation and photography of the H33342 accumulation were performed using a Leica DMi8 inverted fluorescence microscope (Leica Microsystems GmbH, Wetzlar, Germany) with 20× magnification for the DAPI and VIS channels for H33342. Observation of accumulation of MIT was performed using a Leica Application Suite X (Leica Microsystems GmbH, Wetzlar, Germany), with a magnification of 20× for Fluo-Red Y5 and VIS channels.

#### 4.9.2. Short-Term VIS Incubation

Dye accumulation was also examined during a short-term incubation of cell lines with VIS. The cells were seeded at the same concentration as above and cultured for 5 days. The medium was then replaced with one containing 10 µM, 25 µM, or 50 µM VIS and incubated for 1 h. Afterward, we added H33342 (final concentration 0.5 µg/mL) or MIT (final concentration 5 µg/mL) and incubated for 1 h. Subsequently, washing and observation of cell accumulation were performed as described above.

### 4.10. Flow Cytometry Analysis

#### 4.10.1. Long-Term Incubation with VIS

The effect of VIS on BCRP protein activity was also checked by testing the accumulation of fluorescent BCRP substrates inside the cells. Cell lines were seeded at a concentration of 1 × 10^5^ cells/mL in a 6-well plate. After 48 h, cells were cultured in medium supplemented with VIS (10, 25, 50 µM). After 72 h, VIS was washed using cell culture medium, the cells were harvested and suspended in cell culture medium with H33342 (final concentration 0.5 µg/mL) or MIT (final concentration 0.2 µg/mL). Incubation with the dye was carried out for 1 h at 37 °C with shaking (800 rpm). Next, the cells were put on ice and centrifuged at 200× *g* for 5 min at 4 °C and washed twice with ice-cold 50 µM verapamil in PBS. Cells were kept on ice until data collection. Cells untreated with VIS and without H33342 or without MIT dyes served as negative controls. The fluorescence emission was measured at 405 nm or 640 nm using a MACSQuant 10 cytometer. In total, 10,000 events were recorded for each analysis. Analysis was performed using FACS-FlowJo 10.9 software.

#### 4.10.2. Short-Term Incubation with VIS

Additionally, the accumulation of H33342 and MIT dyes was determined using flow cytometric analysis after short-term cell treatment. Cell suspensions (1 × 10^6^ cells/mL in culture medium) were treated with VIS (10, 25, 50 µM) or inhibitor-free medium for 1 h at 37 °C with agitation (800 rpm). H33342 (final concentration 0.5 µg/mL) or MIT (final concentration 0.2 µg/mL) was then added, and the cells were incubated under the same conditions for 1 h. After incubation, cells were put on ice, centrifuged at 200× *g* for 5 min at 4 °C and washed twice with ice-cold 50 µM verapamil in PBS. Cells were kept on ice until data collection. Cells untreated with VIS and without H33342 or without MIT dyes served as negative controls. Data were collected and analyzed as described above.

### 4.11. Live-Cell Fluorescence (H33342 and MIT Accumulation) (3D)

Spheroids were obtained by culturing cells (1 × 10^4^ cells per well) in non-adherent 96-well plates for 5 days. Spheroids were then treated with appropriate concentrations of VIS (10, 25, 50 µM) for 1 h. H33342 (µg/mL) or MIT (2 µg/mL) was added to the spheroids so that the VIS concentration remained unchanged. Spheroids without dyes and inhibitor treatment were used as negative controls. After 1 h, spheroids were washed with cold 50 µM verapamil in PBS. H33342 accumulation was observed under a fluorescence microscope (Leica DMi8) at 10× magnification for the DAPI and VIS channels. In contrast, MIT accumulation was observed under a fluorescence microscope (Leica DMi8) with 20× magnification, for Fluo-Red Y5 and VIS channels.

### 4.12. Assessment of Apoptosis/Necrosis in 3D Conditions

The cells were seeded in non-adherent 96-well plates. After 48 h, when aggregates started forming, proper concentrations of VIS (0, 40, 80, 100, 200, 400, 600 μM) were added. Following 72 h incubation, a mixture containing final concentrations of 10 μg/mL Propidium Iodide, 1 μg/mL Hoechst 33342, and 1 μM elacridar was added to the wells for 2 h. Afterward, the wells were carefully washed with 50 μM Verapamil in PBS three times. Finally, the aggregates were observed and photographed under the Leica DMi8 microscope.

### 4.13. Colony Formation

Cells were seeded into 6-well plates at 500 cells per well. After 48 h of cell culture, the medium was changed to the one containing 0, 10, 25, or 50 µM VIS. After 3 days of cell culture, the medium was replaced with a new one, containing the appropriate inhibitor. After 7 days, the cells were washed with PBS containing Mg^++^ and Ca^++^ and fixed by a 15min incubation with methanol. The formed colonies were then stained with crystal violet (0.5% in 20% methanol) during the 15 min incubation, followed by washing the dye away with running tap water. The plates were scanned, and the area occupied by the colonies was calculated with ImageJ software (1.48q, Rayne Rasband, National Institutes of Health, USA).

### 4.14. Cell Migration—Wound Healing Assay

The cell lines were seeded into 6-well plates at a concentration of 7 × 10^5^ cells per well in 2 mL medium. After 48 h of culture, the medium was replaced with one containing the appropriate concentration of VIS (0, 10, 25, 50 µM). After 24 h, a scratch was made using a pipette tip. The cells were washed twice with PBS, and medium with the appropriate concentration of VIS was added. The scratch was photographed using a Leica DMi8 microscope under visible light at 10× magnification. Images were taken every 24 h for the next four days. Every day before the image was taken, the culture medium was replaced with fresh medium containing the appropriate VIS concentration. Analysis of the wound closure areas was performed using ImageJ software (1.48q, Rayne Rasband, National Institutes of Health, USA) and calculated using the following formula: ([scratch area at time 0 − cell-free area at time n h]/scratch area at time 0 × 100%).

### 4.15. Cell Proliferation Assay

Cells were seeded into 96-well plates at a concentration of 1 × 10^3^ cells per well in a volume of 200 µL of medium. After 48 h, the medium was replaced with a medium containing the appropriate concentrations of VIS (0, 10, 25, 50 µM). At the same time, the MTT assay was performed, as described in the Two-Dimensional MTT Assay section. This measurement was considered the 0 h point. MTT assays were then performed every 24 h for the next 4 days. Cell line proliferation was determined relative to the 0 h point.

### 4.16. Statistical Analysis

Data gathered from the qPCR and MTT assays were analyzed using Student’s t-test and presented as the mean ± standard error of the mean (SEM). A *p*-value of less than 0.05 was considered statistically significant. Each experiment was conducted in a minimum of four independent replicates.

## Figures and Tables

**Table 1:** Summary of cell line resistance to VIS treatment in 2D cell culture conditions. The mean IC_25_ and IC_50_ values for VIS are reported for each cell line, with the range presented in brackets.

Cell Line	IC_25_ [µM]	IC_50_ [µM]
A2780	24.2	46.1
	(18.4–36.0)	(38.4–60.5)
A2780TR1	24.7	51.5
	(21.4–29.6)	(47.5–58.0)
A2780TR2	23.7	42.5
	(15.7–33.1)	(26.8–53.4)

**Table 2:** Summary of cell line resistance to TOP treatment in 2D cell culture conditions in the absence and presence of VIS at concentrations of 10 and 25 µM. The TOP IC_50_ values are indicated for each cell line. The TOP resistance in the cell lines treated with TOP alone was assigned a value of 1 for every cell line. Underlined values indicate multiplicities of sensitivity in the presence of VIS with respect to cells treated with TOP alone. The up/down arrows indicate an increase/decrease in IC_50_ value compared to the control. ** *p* < 0.01, *** *p* < 0.001.

Cell Line	Control TOP IC_50_ (ng/mL)	VIS 10 µM TOP IC_50_ (ng/mL)	VIS 25 µM TOP IC_50_ (ng/mL)
A2780	7.68	7.23	7.94
	(6.17–8.45)	(5.85–8.27)	(5.60–9.08)
	1	1.06 ↓	1.03 ↑
A2780TR1	75.5	17.9	10.2
	(61.4–92.3)	(14.5–22.4)	(7.54–13.0)
	1	4.20 ↓**	7.40 ↓**
A2780TR2	81.1	20.4	13.4
	(71.1–89.4)	(16.9–24.5)	(11.7–15.0)
	1	3.98 ↓***	6.03 ↓***

**Table 3:** Summary of cell line resistance to TOP treatment in 3D cell culture conditions in the absence and presence of VIS at concentrations of 10, 25, and 50 µM. The TOP IC_50_ values are indicated for each cell line. The TOP resistance in the cell lines treated with TOP alone was assigned a value of 1 for each cell line. Underlined values indicate multiplicities of sensitivity in the presence of VIS with respect to cells treated with TOP alone. The down arrows indicate an increase/decrease in IC_50_ value compared to the control. * *p* < 0.05, ** *p* < 0.01, *** *p* < 0.001.

Cell Line	Control TOP IC_50_ (ng/mL)	VIS 10 µM TOP IC_50_ (ng/mL)	VIS 25 µM TOP IC_50_ (ng/mL)	VIS 50 µM TOP IC_50_ (ng/mL)
A2780	8.38	7.78	8.05	8.04
	(7.16–9.28)	(7.04–8.62)	(7.26–8.73)	(7.35–8.59)
	1	1.08 ↓	1.04 ↓	1.04 ↓
A2780TR1	361	68.9	41.0	41.1
	(272–436)	(48.3–89.8)	(26.8–56.6)	(15–93.2)
	1	5.23 ↓**	8.79 ↓**	8.78 ↓***
A2780TR2	319	98.1	39.8	26.3
	(241–364)	(35.6–183)	(9.82–74.6)	(7.98–62.8)
	1	3.25 ↓*	8.00 ↓**	12.1 ↓**

**Table 4:** Summary of cell line resistance to VIS in 3D cell culture conditions. The mean IC_25_ and IC_50_ values for VIS are reported for each cell line, with the range presented in brackets.

Cell Line	IC_25_ [µM]	IC_50_ [µM]
A2780	56.01	107.14
	(21.69–89.19)	(82.80–160.38)
A2780TR1	32.42	86.01
	(18.22–50.00)	(56.18–89.32)
A2780TR2	45.46	92.90
	(14.12–107.26)	(41.25–173.05)

**Table 5:** Comparison of VIS resistance in 2D and 3D cell culture conditions. The VIS IC_50_ values are indicated for each cell line. The VIS resistance for each cell line cultured in 2D conditions was assigned a value of 1. Underlined values indicate multiplicities of sensitivity in 3D conditions with respect to the same cell line cultured as a monolayer. The up arrows indicate an increase in IC_50_ value compared to the control. * *p* < 0.05.

Cell Line	2D IC_50_ [µM]	3D IC_50_ [µM]
A2780	46.13	107.14
	(38.4–60.5)	(82.80–160.38)
	1	2.32 ↑*
A2780TR1	51.46	86.01
	(47.5–58.02)	(56.18–89.32)
	1	1.67 ↑*
A2780TR2	42.54	92.90
	(26.8–53.4)	(41.25–173.05)
	1	2.17 ↑*

**Table 6:** Primers used for qPCR procedures.

Transcript	Sequence (5′–3′ Direction) Forward	Sequence (5′–3′ Direction) Reverse	ENST Number http://www.ensembl.org	Product Size (bp)
BCRP	TTCGGCTTGC- AACAACTATG	TCCAGACACA- CCACGGATAA	00000237612	128
GAPDH	GAAGGTGAAG- GTCGGAGTCA	GACAAGCTTC- CCGTTCTCAG	00000229239	199
ALDH1A1	GTTGTCAAAC- CAGCAGAGCA	CTGTAGGCCC- ATAACCAGGA	00000165092	115
PTPRK	CCCAGGACCT- CCACTAATCA	ATTCCCAGTC- CACAGCAATC	00000368226	110
SMO	TACGTCAATG- CGTGCTTCTT	CGCAGGACAG- AGTCTCATTG	00000249373.8	139
PCTCH1	GGCACAGTCA- AGAACAGCAC	TGTCCTCGTTC- CAGTTGATG	00000430669.6	139
SHH	TCCAAGGCAC- ATATCCACTG	CTCAGGTCCT- TCACCAGCTT	00000297261.7	128
ILK	TGTCGTGAAG- GTGCTGAAGG	ATGAGGAGCA- GGTGGAGACT	00000299421.9	142
GLI1	ACACGGGTGA- GAAGCCATAC	GCAGCCAGGG- AGCTTACATA	00000228682	134
GLI2	TGGAGCACTA- CCTCCGTTCT	CCCCTCTCCT- TAAGGTGCTC	00000361492	109
GLI3	GCTCTCCATG- ATCTCAGCAA	GGCAGCTGAG- GGAATAATGT	00000395925.8	142

## Data Availability

The data presented in this study are openly available in [https://repod.icm.edu.pl/dataset.xhtml?persistentId=doi:10.18150/MS6ZTY], accessed on 22 May 2026 at [doi:10.18150/MS6ZTY].
